# Effective Oxidation State Analysis for Solids

**DOI:** 10.1021/acs.jctc.5c00482

**Published:** 2025-07-03

**Authors:** Gerard Comas-Vilà, Leila Pujal, Alberto Otero-de-la-Roza, Davide Tiana, Julia Contreras-Garcia, Pedro Salvador

**Affiliations:** † Institut de Química Computacional i Catàlisi i Departament de Química of Computational Chemistry and Catalysis, Chemistry Department, 16738 University of Girona, Montilivi Campus, Girona, Catalonia 17003, Spain; ‡ Department of Chemistry, 4257 Queen’s University, 90 Bader Lane, Kingston, Ontario K7L-3N6, Canada; § Departamento de Química Física y Analítica and MALTA-Consolider Team, Facultad de Química, Universidad de Oviedo, 33006 Oviedo, Spain; ∥ School of Chemistry, University College Cork, College Road, T12 K8AF Cork, Ireland; ⊥ Laboratoire de Chimie Théorique (LCT), Sorbonne Université, CNRS, 4 Place Jussieu, 75005 Paris, France

## Abstract

We present the generalization of the effective oxidation state (EOS) method to assign oxidation states from wave function analysis to solid-state calculations. The scheme is realized in the framework of the Quantum Theory of Atoms in Molecules (QTAIM), and makes use of the atomic overlap matrices (AOM) of the atoms of the unit cell, expressed (whenever possible) in terms of maximally localized Wannier functions (MLWFs). The method is generally applicable to ionic solids or molecular crystals. The performance of the new method is assessed using a chemically diverse set of 40 solids, including simple metal oxides, perovskites, hydrides, and high-pressure systems with unusual bonding patterns.

## Graphical abstract

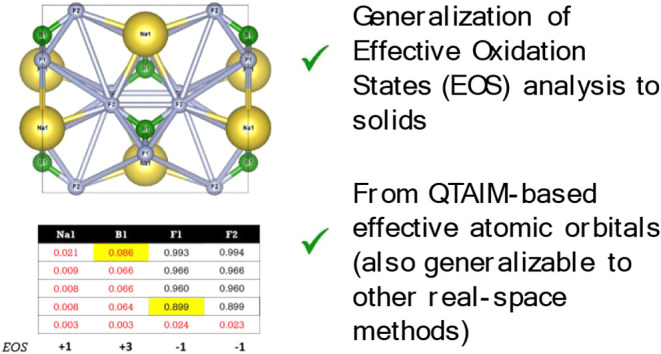

## Introduction

The concept of electric charge associated with a specific atom or ion has been fundamental to chemistry since Michael Faraday’s work on electrolysis in 1834. Faraday’s laws established that charge transfer between electrodes is always an integer multiple of the elementary electronic charge and is directly proportional to the number of atoms exchanged – a phenomenon rooted in the fundamental quantization of charge.

In the context of static structures, the concept of atomic oxidation states (OS) was later formalized by Wendell Latimer in 1938,^1^ providing a framework to describe the formal charge on an atom based on a set of agreed-upon rules. While the OS of an atom in a molecule cannot be directly measured, spectroscopic techniques provide indirect methods to infer OS. These techniques rely on the fact that OS changes alter an atom’s electronic environment, leading to distinct spectral patterns. For example, in X-ray Absorption Near Edge Structure (XANES), higher oxidation states typically result in higher absorption edge energies due to the increased effective nuclear charge. Extended X-ray Absorption Fine Structure (EXAFS) provides information about the local coordination environment, which can also be influenced by the OS. Another powerful technique is Mössbauer Spectroscopy, which measures the resonant absorption of γ rays by nuclei, such as ^57^Fe. The isomer shift, a key parameter in Mössbauer spectra, is directly related to the electron density at the nucleus. Higher OS generally results in lower electron density and thus a larger isomer shift. Additionally, quadrupole splitting provides information about the symmetry of the electron distribution around the nucleus, which can also be influenced by the OS.

It is important to bear in mind that these techniques assign OS by comparing the spectral features of a sample to those of reference compounds with *known* OS, assuming that the electronic and structural environment of the reference compounds is sufficiently similar to the sample being studied. However, if suitable reference compounds are not available, or if the sample has a unique electronic or structural environment that differs significantly from the reference, the assignment of OS can become ambiguous or inaccurate. For example, in XAS, the absorption edge energy can be influenced not only by OS but also by coordination geometry and ligand effects, and in Mössbauer spectroscopy, the isomer shift also depends on the spin state of the atom.

Thus, while experimental techniques provide valuable insights into OS, their reliance on reference compounds and the influence of additional factors can introduce uncertainties.

In 2014, the International Union of Pure and Applied Chemistry (IUPAC), revised the concept of OS, providing a new (improved) generic definition.^2^ In a nutshell, OS is defined as the hypothetical charge the atom would carry if all its bonds were purely ionic. As such, IUPAC’s straightforward definition is widely used by chemists to assign OS and predict material properties. Since different OS of the same element often exhibit distinct physical characteristics (ionic radii, shifts in X-ray emission spectra) accurate OS predictions can help interpret a material’s structural, electronic, optical, and magnetic behavior. This predictive power underscores the need for reliable schemes/algorithms to assign OS. The IUPAC’s technical report^3^ provided back-of-the-envelop algorithms to assign OS for molecules and solids, but exceptions, ambiguities, and limitations have already been exposed.^4^ All in all, situations where the assignments of OS become ill-defined are usually those associated with intriguing new physics or chemical properties. In this scenario, computational techniques specifically devised to extract oxidation states from wave function analysis play a fundamental role.

The elucidation/assignment of oxidation state in solid-state physics is not as straightforward as in insulating liquids for two reasons. On the one hand, it requires attributing the electrons to the atoms from quantum computations (which does not have an unambiguous definition). On the other hand, and unlike the quantized charges observed in electrolysis, oxidation states in quantum systems lack a clear quantization theorem. Whereas nuclei travel over macroscopic distances in liquid insulators,^5^ this is not the case of crystalline insulators. The wave function does not inherently partition into discrete atomic contributions, particularly when electron density overlaps between neighboring atoms.

These two issues were elegantly and simultaneously solved by Rappe^6^ and extended by Resta^7^ in crystalline systems. Since integer charges manifest themselves in the calculation only when transported they implemented cell-wise sublattice movements with localized Wannier functions. When an atom moves to its image position in periodic insulators, the change of polarization reveals the number of Wannier function centers that move with the atom, provided that the system stays insulating. This effectively indicates the number of electrons that *belong* to the nucleus and establishes a rigorous way of elucidating the oxidation state of ions in solids. This was shown to work for prototypical examples such as LiH, but also for metallic atoms with various oxidation states. However, it requires several single-point calculations since the sublattices need to be displaced.

Other approaches have been introduced tackling the two challenges (atomic attribution and integer rounding) separately; which have not been devoided of detractors.^8,9^ As far as atomic attribution is concerned, the classical approach in solid-state physics has been to carry out a geometrical partitioning using Voronoi cells, where the charge within a weighted polyhedron is associated with an atom. As in molecules, Hilbert-space and real-space partitions have also been proposed, which then need a posteriori treatment for obtaining integer OS.

Within Hilbert space approaches, analogous to Mulliken partitions, it is possible to project the wave function (WF) to localized atomic orbitals. However, this approach suffers from dependence on the basis set. In order to then produce integer OS, further projections have been proposed for metal–ligand interactions: electrons are attributed (or not) to the metal center depending on whether the metal–ligand interaction implies electron donation (or not). This attribution avoids the negative feedback observed in interactions which tends to compensate charges^10,11^ but does not work when a strong metal–metal bond is present or one is interested in the OS of a particular ligand.^12^

As for real space partitionings, topological approaches have been commonly used to define atoms in a molecule. Probably the most well-known among them is the topological analysis within the Quantum Theory of Atoms in Molecules (QTAIM),^13^ which partitions electron density into atom-centered basins. However, as expected from the delocalized nature of the wave function, once again these partitions yield fractional atomic charges, which are then rounded to achieve integer OS. A noticeable (and controversial example) was that of TiO_2_. QTAIM charges lead to Ti^+2.5^ which was rounded to Ti^3+^,^14,15^ in contrast with the expected OS + 4 according to IUPAC rules. This stems from the fact that there is an appreciable electron density in the Ti 3d orbitals due to bond polarization, which leads to a smaller QTAIM charge.^10^

Another option based on real space topological partitioning present in the literature is ELIBON (Electron Localization Indicator-based oxidation numbers). The Electron Localization Indicator function provides the electron localization regions in a solid. To retrieve an atomic attribution, the electrons from bonds are attributed to atomic centers from an electron density partitioning, whereas lone pairs are attributed to the atom hosting them (typically some corrections to the core population are also carried out^16^). This partitioning also leads to fractional charges, which are then rounded.^17,18^ Most recently, mixed geometrical/topological schemes have also been introduced to account for specific materials, such as electrides.^19^

Although polarity is well taken into account in the ELIBON approach, rounding remains a problematic choice in all these real-space approaches: if adjacent atoms are assigned charges of 1.5 and 2.5 electrons, how should the excess electron be distributed? These issues mainly stem from a conceptual definition. Indeed, the challenge of determining OS from first-principles has been significantly hampered by the implicit assumption that partial atomic charges either determine or are closely related to OS. Partial atomic charges represent the (noninteger) *average* number of electrons associated with an atom based on a specific electron-partitioning scheme. In contrast, oxidation states are integer values representing a conceptual, fictitious charge assigned to an atom under the ionic approximation formalism. This distinction is crucial because partial atomic charges reflect a continuous distribution of electron density, while oxidation states are discrete and idealized.

This quandary was solved within the molecular realm some years ago with the so-called effective oxidation state (EOS) analysis, a general scheme applicable both to Hilbert and real space partitionings and even beyond single-determinant WFs.^20^ However, its implications for solid-state materials remain largely unexamined. In this article, we extend the methodology developed for molecular systems to the solid-state domain, offering a new approach to assigning and interpreting oxidation states in crystalline materials. Through case studies on a range of solid-state structures—including examples from IUPAC’s technical report but also more exotic materials such as mixed-valence, correlated systems, and electrides. In the latter case, we highlight the limitations imposed by abnormal QTAIM topologies, shedding light on the broader challenges of defining oxidation states in quantum systems.

## Theory

In 2015, Ramos-Cordoba et al.^20^ introduced a new and general scheme to derive OS from wave function analysis of molecular electronic structure calculations. The method is formally applicable on equal footing to any molecular system and for any level of theory.^21^ The so-called effective oxidation state (EOS) analysis relies on the occupation number of the so-called spin-resolved effective fragment orbitals (EFOs), a generalization to molecular fragments of Mayer’s effective atomic orbitals (eff-AOs), obtained for all atoms or molecular fragments defined. In the process of assigning the OS, the EFOs are considered as either occupied or empty, leading to an effective configuration of these atoms or fragments within the molecular system, which directly determines their OS. The EFOs can be obtained in the framework of Hilbert-space or real-space analyses. In the particular case of a single-determinant WF and the QTAIM framework, they are simply obtained by diagonalization of the atomic/fragment overlap matrices (AOM) in the MO basis for each spin-channel σ (α or β), with elements

1 $$S_{\mathrm{ij}}^{\sigma ,A}=\int_{\Omega_{A}}\phi_{i}^{\sigma ,*}\left(r\right)\phi_{j}^{\sigma }\left(r\right)\mathrm{dr}$$

where Ω*_A_* represents the physical domain of the atom or fragment. The diagonalization of ***S*** ^σ,*A*^ by the unitary matrix ***U*** ^σ,*A*^

2 $$U^{\sigma ,A+}S^{\sigma ,A}U^{\sigma ,A}=\Lambda^{\sigma ,A}=diag\left\{\lambda_{i}^{\sigma ,A}\right\}$$

yields at most *n*_occ_^σ^ eigenvalues with λ_*i*_^σ,*A*^ ≥ 0, where *n*_occ_^σ^ is the number of occupied MOs of spin channel σ. For each atom or molecular fragment *A* we can define *n*_*A*_^σ^ ≤ *n*_occ_^σ^ *effective* atomic/fragment orbitals χ_μ_^σ,*A*^(***r***) as linear combinations of the MOs, but defined only within the respective domain Ω*_A_*

3 $$\chi_{\mu }^{\sigma ,A}\left(r\right)=\frac{1}{\sqrt{\lambda_{\mu }^{\sigma ,A}}}\sum_{i=1}U_{i\mu }^{\sigma ,A}\phi_{i}^{\sigma }\left(r\right),\mu =1,2,...,n_{A}^{\sigma }.,\mathrm{where}\;r\in \Omega_{A}$$

The occupation number of each EFO is given by the eigenvalues 0 ≤ λ_μ_^σ, *A*^ ≤ 1. The sum of the occupation number of the *n*_*A*_^σ^ EFOs recovers the population of the atom/fragment *A* for the given spin case

4 $$N^{\sigma ,A}=\sum_{\mu }\lambda_{\mu }^{\sigma ,A}$$

In the case of atomic domains, the shape and occupation number of the eff-AOs faithfully reproduce the core and valence shells; those with occupation numbers close to 1 are associated with core orbitals or lone pairs, whereas those with smaller but significant occupation are identified with the atomic orbitals directly involved in the bonds. The remaining EFOs are marginally occupied and have no chemical significance.

The EOS analysis algorithm, depicted in Scheme 1, goes as follows: (i) the α EFOs are obtained and collected for all atoms/fragments, (ii) the EFOs are sorted according to decreasing occupation number, and (iii) integer α electrons are assigned to the EFOs of the atoms/fragments with higher occupation number until the number of α electrons is reached. Then, proceed analogously for the β electrons if needed (in a restricted closed-shell WF the eff-AOs of both spin channels are identical). By this procedure, an effective electronic configuration is obtained for each atom/fragment. The OS of each atom/fragment is simply given by the difference between its atomic number and the number of α and β electrons that have been assigned to it. This scheme can be safely applied to basis sets including effective core potentials, simply by readily assigning the electrons described by the atomic core potential to the given atom.

**Scheme 1:**
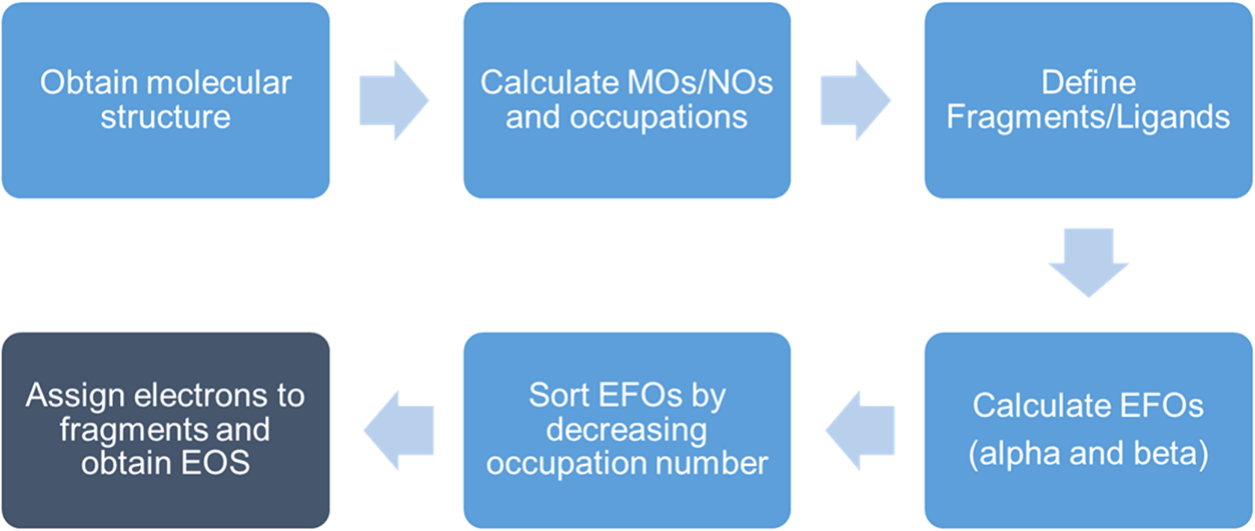
Effective Oxidation State (EOS) Analysis Scheme Flowchart

It is important to note that the occupation numbers of the EFOs are not simply rounded to the closest integer. Instead, they are sorted in decreasing order for each spin case, and the first *n* ^σ^ EFO are considered occupied, being *n* ^σ^ the total number of electrons for spin case σ. By comparing the occupation numbers, the effects of applying one or another atom-in-molecule (AIM) scheme to obtain the EFOs are minimized. Such a strategy also underlines the fact that the OS depends on all atoms of the system and of course on the total number of electrons. Hence, in the case of electron-deficient systems, it is possible to have the last EFO considered as occupied by the EOS algorithm with an occupation smaller than 0.5. Contrarily, electron-rich systems such as formal Cu­(III) complexes with CF_3_ ligands can exhibit unoccupied EFOs with occupation numbers larger than 0.5.^22^

The occupation numbers of these frontier EFOs, namely the last occupied, λ_LO_^σ^, and the first unoccupied, λ_FU_^σ^, can be used, for each spin case, to indicate how close the formal picture given by the OS is to the actual electronic distribution of the system. When λ_LO_^σ^ and λ_FU_^σ^ differ by more than half an electron (i.e., a full electron rounding up the difference in occupation number) the assignment of EOS is considered as indisputable. For each spin case, the reliability index *R* ^σ^(%) reads

5 $$R^{\sigma }\left(\%\right)=100\min\left(1,\max\left(0,\lambda_{\mathrm{LO}}^{\sigma }-\lambda_{\mathrm{FU}}^{\sigma }+1/2\right)\right)$$

and then *R*(%) = min­(*R* ^α^(%), *R* ^β^(%)). That is, the overall *R*(%) index is the minimum value obtained for either the α or β electrons. The larger the *R*(%) value the closer the overall assignment of the EOS is to the actual electronic structure of the system. Note that *R*(%) can take values formally from 0 to 100, where values below 50% indicate that the assignment of the electrons has not followed an Aufbau principle according to the occupation numbers of the EFOs. The latter avenue can be used to measure to which extent the system conforms with a given set of oxidation states, rather than which are the most appropriate formal oxidation states.

If the frontier eff-AOs for any spin case are degenerated (same occupation number) and belong to different fragments, a value of *R* = 50% would be obtained. In that case, however, one may choose to assign a half-electron to each of the two atoms/fragments involved (or, in general, a fraction of the last *m* electrons that must be distributed among *n*_d_ degenerated EFOs). We use such an approach only when the degeneracies are due to symmetry. Alternatively, one might define a (small) threshold to consider two or more EFOs as pseudodegenerated when their occupation numbers are close enough.

Finally, it is also important to stress that the reliability index does not measure any probability. A given OS assignment with *R* = 65% does not necessarily imply that it is 65% likely to be the “right” assignment. Instead, it should be considered as a similarity measure between the formal ionic picture and the actual electron distribution. As such, the particular expression given in eq 5 is to some extent arbitrary. What holds greater significance is identifying the frontier EFOs, understanding their shapes, and analyzing how their respective occupations compare.

Mayer et al.^23,24^ showed that the eff-AOs and their occupation numbers can be obtained in the framework of real-space analysis even in the absence of an underlying atom-centered basis set. They obtained the eff-AOs in the framework of *fuzzy atoms* from numerical MOs (e.g., cube files) obtained for molecular systems described with plane waves. The results were very close to those obtained for molecular LCAO calculations. Motivated by their findings, this work aims to extend the aforementioned EOS analysis scheme to condensed phase calculations.

## Results and Discussion

To apply the EOS analysis to solid-state calculations, the AOMs obtained with an underlying AIM definition (eq 1) are required. In this work, we basically follow the steps described by Otero-de-la-Roza et al. to evaluate localization/delocalization indices in solids.^25^ The proper topological analysis of the electron density in a pseudopotentials/plane-waves approach requires all-electron densities. These are reconstructed using projector-augmented-wave (PAW) calculations. A conventional norm-conserving pseudopotential calculation is then used to obtain the Bloch states. The AOMs expressed in terms of maximally localized Wannier functions (MLWFs) are obtained in the framework of QTAIM for all atoms (attractors) in the unit cell (see Computational Details). The corresponding eff-AOs for each atom in the unit cell are obtained by diagonalization of the AOMs, and their occupations are given by the eigenvalues. In the case of fragments within the unit cell, the overlap matrices are obtained simply by the addition of the AOMs of the atoms forming the fragment. It is important to note that this is possible because the atomic domains are disjoint (nonoverlapping). Once the eff-AO occupations are obtained, the conventional EOS algorithm outlined in Scheme 1 can be applied.

One important caveat must be considered when determining the reliability index *R*(%). In the EOS analysis for molecular systems, the frontier EFOs must belong to different fragments. If they were from the same fragment, selecting the first unoccupied orbital instead of the last occupied orbital would leave the OS assignment unchanged. However, this condition does not apply to solids. To illustrate, consider a binary solid AB with two atoms in the unit cell. Atom A hosts both the last occupied and first unoccupied eff-AOs, with occupations of 0.6 and 0.4, respectively. The next unoccupied eff-AO belongs to atom B, with an occupation of 0.2. The EOS scheme for molecules would calculate the reliability index using the eff-AOs with occupations 0.6 and 0.2, yielding *R*(%) = 90.

However, if a supercell approach is employed (e.g., using two unit cells), two sets of identical eff-AOs would arise for symmetry-equivalent atoms A (A’) and B (B’). Since the number of electrons to distribute also doubles, the eff-AO with an occupation of 0.6 would be formally occupied for both A and A’, while the eff-AO with an occupation of 0.4 would be formally unoccupied. In this scenario, the last occupied eff-AO could be associated with center A, and the first unoccupied eff-AO with center A’. Consequently, the reliability index would be determined using the eff-AOs with occupations 0.6 and 0.4, resulting in a different value for *R*(%). Hence, to avoid having different *R*(%) values for essentially the same electronic structure, the EOS scheme for solids allows the frontier eff-AOs to be associated with the same atom or fragment.

We utilized the EOS method to study over 40 solids listed in Table 1. These compounds can be classified into different categories. The first compounds (**1–15**) were considered for being included as illustrative examples of the algorithm of assigning bonds, discussed in the IUPAC technical report.^3^ Within this category, the compounds can be further divided into subsets, such as metal oxides, perovskites, inverse perovskites, and transition metal complexes. Also, we have analyzed some metal hydride structures (**16–23**)^26^ containing hydridic hydrogens and molecular hydrogen units. Finally, we test the limits of applicability of the method considering high-pressure compounds where unusual bonding patterns and oxidation states are suggested leading to *novel* chemical phenomena. In this group, we can find xenon oxides where the possibility of gaining or losing electrons for the noble gas atom becoming an anion or cation has been discussed (**24–33**).^27,28^ Also, the potential activation of the 5d core electrons in the HgF_4_ compound has been proposed (**34–36**)^29^ going beyond the common OS + 2 for the Hg atom. Finally, systems containing main-group elements exhibiting homonuclear bonds are also considered (**37–40**).^30–32^ For the first 15 compounds, we validate our OS assignments with that provided by IUPAC’s algorithms and recommendations. In most cases, it consists of the application of the ionized bond order sum (IBOS) algorithm, particularly suitable for bond graphs (i.e., extended solids).^3^ In the other systems studied, our assignment is confronted with existing assignments derived from partial charges, electron localization function (ELF) shape, or geometric criteria.

**Table 1:** Chemical Formula of All Systems Considered in This Work^a^

ID	chemical formula	reference	ID	chemical formula	reference
1	CrO_3_	COD 1535017	21	ZrH_4_	ref[^26^]
2	KMgF_3_	CCDC 1610327	22	CeH_4_	ref[^26^]
3	NaBF_4_	CCDC 1607866	23	ThH_4_	ref[^26^]
4	SrBe(OH)_4_	CCDC 784604	24	XeO	ref[^27^]
5	Cs_3_AuO	CCDC 1639348	25	XeO	ref[^27^]
6	Rb_3_AuO	CCDC 1636148	26	XeO_2_	ref[^27^]
7	Ca_3_AuN	CCDC 1633769	27	XeO_2_	ref[^27^]
8	K	ref[^33^]	28	XeO_3_	ref[^27^]
9	GaSe	CCDC 1626255	29	XeO_3_	ref[^27^]
10	Ba_3_Si_4_	CCDC 1732102	30	XeF_2_	CCDC 1602015
11	Cs_2_Pt	CCDC 1728284	31	XeF_3_	CCDC 1597685
12	WCl_4_	CCDC 1681327	32	XeF_4_	CCDC 1602016
13	Rb_2_CuCl_4_	CCDC 1595922	33	MgXe	ref[^28^]
14	YBaFe_2_O_5_	CCDC 1721776	34	HgF_4_	ref[^29^]
15	YBaFe_2_O_5_	CCDC 1721780	35	HgF_4_	ref[^29^]
16	MgH_4_	ref[^26^]	36	HgF_4_	ref[^29^]
17	CaH_4_	ref[^26^]	37	CsH_3_	ref[^30^]
18	SrH_4_	ref[^26^]	38	CsF_3_	ref[^31^]
19	ScH_4_	ref[^26^]	39	CsF_5_	ref[^31^]
20	YH_4_	ref[^26^]	40	LiN_5_	ref[^32^]

a COD: Crystallography Open Database, CCDC: Cambridge Crystallographic Data Center.

The results of the EOS analysis are gathered in Tables S1–S16 of the Supporting Information jointly with the names, formulas, and figures of all systems indicated by bold numbers in parentheses in this work. Let us start the discussion with the simple binary oxide CrO_3_ (**1**). The computed OS for the central metal atom is +6, which agrees with the chemically expected value also reported by the IUPAC. Although this OS assignment is quite straightforward with the direct ionic approximation, determining a very high oxidation state using theoretical methods is not that simple. The QTAIM partial charge for Cr is only +1.76, very far from the formal +6 value. This illustrates once again that partial atomic charges (disregarding the atomic partitioning used) should not be put into correspondence with OS. Notably, even though the large difference in the partial charge and formal charge, the reliability index of the EOS assignment is quite high (*R*(%) = 71), indicating a quite clear OS assignment. In the case of the set of perovskites (**2–4**), EOS analysis yields the expected outcome, namely K^+^Mg^2+^(F^–^)_3_, Na^+^B^3+^(F^–^)_4_, and Sr^2+^Be^2+^(OH^–^)_4_, with maximum values of the reliability index in all cases. In the latter we have illustrated the use of molecular fragments for the analysis, in this case considering OH units.

The OS assignment in the case of the inverse perovskites Cs_3_AuO (**5**) and Rb_3_AuO (**6**) is also unambiguous, with *R­(%*) = 100, fully consistent with the IUPAC report. However, it is important to note that perovskite **6** exhibits metallic behavior. While the ionic approximation may be questioned in this context, the only impact on the EOS analysis is that it precludes the use of MLWFs. Instead, the AOMs must be obtained directly from the Bloch states, making its evaluation less efficient and more computationally demanding.

The also metallic Ca_3_AuN perovskite (**7**) deserves particular attention. Karen et al.^3^ noted that applying the same strategy used for the other perovskites would lead to unexpected formal Au (−3) anions, breaking the 12-N rule (i.e., atoms close to having 12 *dsp* electrons in their valence shell tend to either lose or gain electrons to obtain an electronic configuration of 12 *dsp* electrons). Therefore, they conclude that this system could be more accurately characterized as Au (−1) + 2e^–^, displaying electride-like properties.

EOS analysis assigns 7 valence α and 7 valence β electrons to the Au center, making it a formal anionic Au (−3) center (see Table S3). The metallic nature of the compound leads to strong electron delocalization, which manifests as a significant number of eff-AOs with relatively low occupation, particularly on the Au centers. The occupation of the last occupied eff-AO on Au is as low as 0.248, with additional eff-AOs on Au showing occupations of 0.230 and 0.218. These compete with an eff-AO on Ca centers with an occupation of 0.144. As a result, the *R*(%) value is quite low at 60.4, indicating a rather ambiguous OS assignment. The alternative view postulated by Karen et al.^3^ can only be supported within our framework if a non-nuclear attractor (NNA) of the density was found. In that case, the NNAs would be considered as additional basins in the unit cell, with associated electron population and AOM, and the EOS analysis algorithm would decide the number of electrons formally assigned to the NNA. However, no non-nuclear attractor could be found from the topological analysis of the density for **7**, at least at the level of theory used. Thus, all these findings indicate that the system exhibits a metallic character with 2 electrons highly delocalized over the cell.

At this point, we have decided to analyze the situation in the case of the well-known high-pressure potassium electride (**8**). According to the topological analysis of the density, the unit cell contains four potassium atoms, and two NNAs are located in the interstitial regions, as illustrated in Figure 1. The presence of NNAs strongly suggests that the solid qualifies as an electride, but the final OS assignment is performed by EOS analysis, treating the K and NNA basins on equal footing. Table S3 shows that the occupation of the first eff-AOs on each K1, K2, and NNA are 0.127, 0.084, and 0.259, respectively. Following EOS analysis, only the eff-AO on the NNA becomes occupied, leading to a formal assignment of two electrons (one α and one β) to each NNA and a formal (+1) oxidation state for each K center. The reliability index for this assignment is low, consistent with the metallic nature of the electride and its pronounced electron delocalization. However, the situation is not as simple in other systems described in the literature. For example, in Ca_2_N, which is characterized as a highly delocalized anionic electride, we have observed that the presence (and number) of interstitial NNAs in the unit cell is highly sensitive to the number of k-points and the grid size used for the topological analysis of the electron density. In this context, Weaver et al.^19^ recently proposed a mixed approach that combines the electron localization function (ELF) with Voronoi polyhedra to identify electride behavior, bypassing the need to explicitly consider the presence or absence of NNAs in the electron density. Such type of real space partitionings might be more suitable, in combination to EOS analysis, to treat electride systems than the current QTAIM based approach.

**Figure 1:**
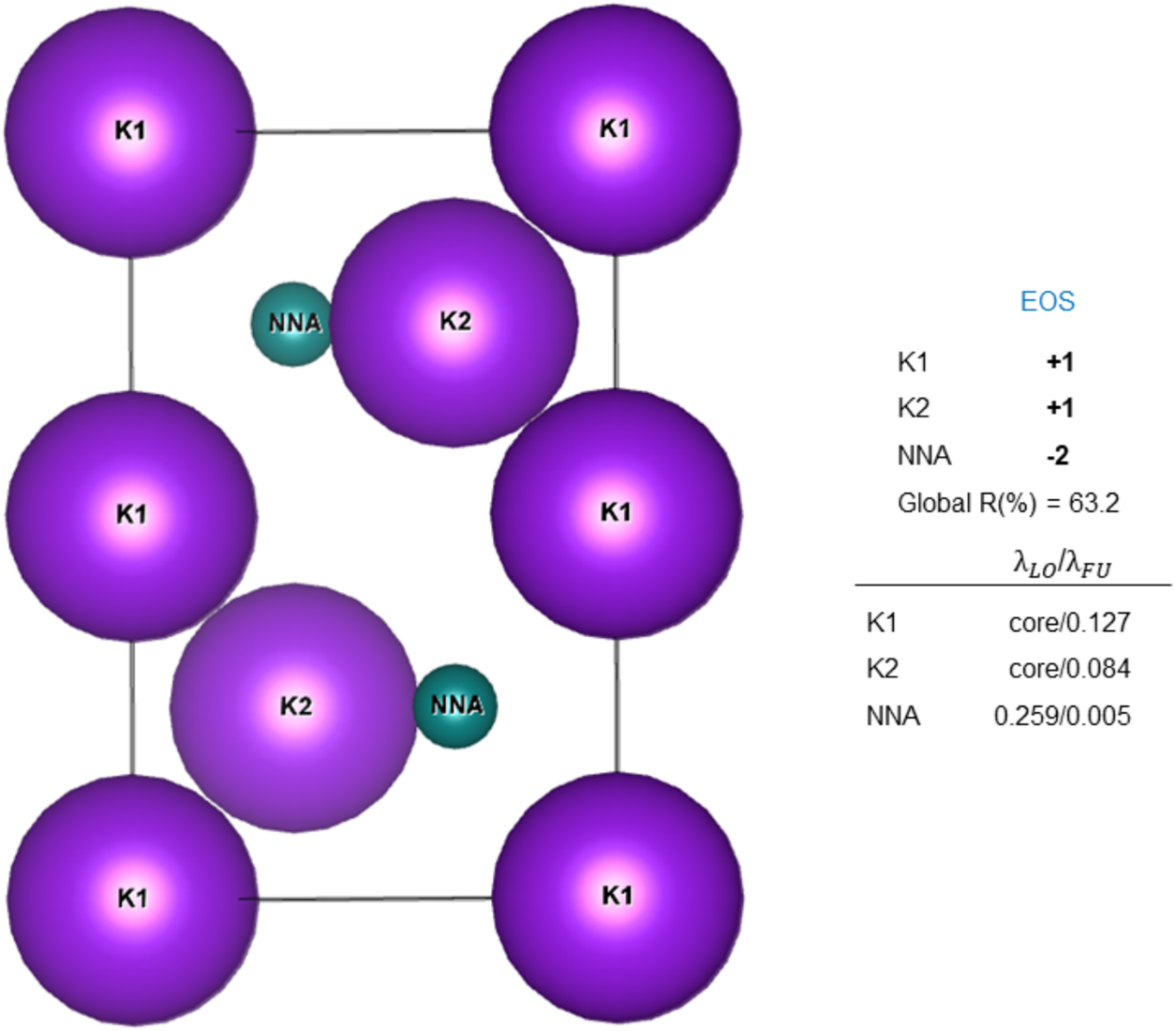
Unit cell for high-pressure potassium electride (**8**) and results of the EOS analysis using atomic fragments. In each case, the occupation of the frontier eff-AOs (λ_LO_/λ_FU_), the *R*% index of the assignment, and the assigned OS are reported.

Another illustrative example is the nonmetallic GaSe compound (**9**) where nine valence electrons must be distributed among the two centers. Figure S4 illustrates the crystal structure of the GaSe unit cell, which adopts a hexagonal arrangement and belongs to the *P*6_3_/*mmc* space group. The unit cell consists of four Ga atoms and four Se atoms. One can clearly identify a Ga–Ga bond and each Se atom is bonded to three Ga atoms. Since there are four GaSe units in the unit cell, there are 36 valence electrons (18 α and 18 β) to assign. Upon application of the EOS scheme, each of the Se centers exhibits four α and four β occupied eff-AOs, being their last occupation of 0.706. The remaining two α and two β electrons must be distributed among four symmetrically equivalent eff-AOs of the Ga centers, with an occupation of 0.581 each. In the undesirable scenario of degenerate frontier eff-AOs, the direct application of eq 5 results in a *R*(%) = 50, and a completely ambiguous OS assignment. However, since the degeneracy in the occupations is forced by symmetry, and there is a Ga–Ga bond, one can opt for assigning half alpha and half-β electrons to each of the four eff-AOs, resulting in the expected Ga­(+2)-Se(−2) OS assignment. An alternative is to consider the Ga–Ga units as a fragment within the EOS algorithm. In this case, each Ga–Ga unit exhibits two meaningfully occupied EFOs, with occupations 0.916 and 0.429. The last two α and β electrons are assigned to the former, while the latter becomes the first unoccupied EFO. The result is a formal Ga–Ga (4+) unit and the same Se (2-) centers, with a much higher *R*(%)= 77.7 value. Thus, the value of the reliability index can be used to justify the formal preference of such Ga_2_-(Se)_2_ picture over the atom-wise one.

Combining sp elements leads to crystal structures with diverse polyanions covalently bonded obeying the Zintl concept.^34^ Among them, the Ba_3_Si_4_ compound (**10**) has been widely studied due to its unexpectedly weakly metallic behavior crystallizing in the space group *P*4_2_/*mnm* with four formula units in the tetragonal unit cell forming butterfly shaped Si_4_^6–^ anions and Ba^2+^ cations according to the IUPAC report. From the EOS perspective and defining each [Si_4_] cluster as a fragment, we clearly reproduced the same OS assignment of Ba­(+2) and Si_4_ (−6) with *R*(%)= 100.

The last series of solids analyzed in the IUPAC’s technical report involve transition metals (**11–15**). We start the discussion with the red transparent Cs_2_Pt platinide (**11**) described as containing closed-shell Pt^2–^ anions. Our EOS scheme gives an undisputable OS assignment consistent with a formal Pt(−2) species in which 6 α and 6 β valence electrons are assigned to the Pt atom, leading to a formal effective electron configuration of 5d^10^6s^2^, fulfilling the 12 *dsp* electrons rule. The WCl_4_ polymer (**12**) was discussed in particular detail. As shown in Figure S6, it exhibits alternate W–W bonds along a chain of edge-sharing octahedra. Being a binary compound, one could be tempted to apply the so-called direct ionic approximation (DIA) and assign the valence electrons to the more electronegative Cl centers. However, these are not strictly symmetry-equivalent. The authors then perform a sophisticated analysis of bond distances and expected bond orders to arrive at the same final W­(4+) and Cl­(1-) assignment using the iBOS algorithm. EOS analysis, on the contrary, leads to the same clear-cut OS assignment with *R*(%) = 92.6, in a more straightforward and unsupervised fashion.

The last two examples correspond to spin-polarized TM systems. In Rb_2_CuCl_4_ (**13**) the iBOS algorithm assuming ideal bond-orders distributes the total 41 valence electrons yielding Cl(−1), Cu­(+2), and Rb­(+1) OS. Let us analyze in detail the EOS assignment process for this species. Since this system is spin-polarized, the α and β spin channels of the density are different, and hence the corresponding eff-AOs. For the α part, 21 electrons must be distributed among the α eff-AOs. The EOS procedure on the α channel unambiguously assigns five 3d electrons to the Cu center and four electrons to each of the four Cl atoms. In the β channel, four 3d β electrons to the Cu and four β electrons to each of the four Cl centers are assigned, for a total of 20 β electrons. The first unoccupied eff-AO is a 3d-type orbital on the Cu atom with a significant occupation (0.496), which nevertheless results in a quite high *R*_β_(%) = 75.4 value. These results emphasize the fact that eff-AO occupations (and populations in general) should not be rounded but compared among the competing ions. Considering all the occupied eff-AOs, the formal electron configuration of the transition metal Cu atom is [Ar]­3d^9^4s^0^.

A final example is the solid YBaFe_2_O_5_ (**14**-**15**), where tautomeric OS transitions occur as a function of temperature. Upon cooling (**14**), two distinct iron centers can be identified. The ionized bond valence sum (BVS) model values, calculated from experimental bond distances,^3,35^ reveal that the two iron atoms approach oxidation states of +2 and +3. The Fe­(+3) site retains a relatively symmetrical coordination environment, while the Fe­(+2) site undergoes significant distortion. At high temperatures (**15**), Mössbauer spectroscopy suggests the presence of only a single iron site, interpreted as a mixed-valent Fe­(+2.5).

The crystal exhibits ferromagnetic behavior, characterized by high-spin iron centers. The EOS analysis clearly assigns five 3d α electrons to each Fe center, with eff-AO occupations exceeding 0.9, as shown in Table 2. However, the electron distribution in the β spin channel is less straightforward, as numerous 3d-type eff-AOs of the Fe centers show significant occupation. When the EOS algorithm is applied to the β spin channel, the last occupied eff-AO is attributed to Fe2, with an occupation of 0.338, while the first unoccupied eff-AO is located on Fe1, with an occupation of 0.282. This results in a closely contested OS assignment, reflected by an *R*(%) value of 55.6. Nevertheless, the preferred OS distribution aligns with the BVS model, assigning Fe1 (3+) and Fe2 (2+).

**Table 2:** Partial Charge (Q), Atomic Spin Density (ρ^s^) and Occupations of the First (3d) Valence Eff-AOs for the Fe Centers in Compounds **14** and **15**

compound	Ion	Q	ρ^s^	spin					
(**14**)	Fe1	+1.40	+3.79	up	**0.950**	**0.950**	**0.950**	**0.943**	**0.939**
				down	*0.282*	0.222	0.206	0.192	0.151
	Fe2	+1.30	+3.72	up	**0.955**	**0.953**	**0.952**	**0.951**	**0.941**
				down	**0.338**	0.266	0.234	0.180	0.136
(**15**)	Fe1	+1.36	+3.77	up	**0.954**	**0.953**	**0.951**	**0.945**	**0.944**
				down	**0.318** ^a^	*0.205*	0.194	0.182	0.179

a Formal split of the last electron pair among two identical Fe centers.

In the high-temperature structure (**15**), the EOS analysis for the α part is quite similar to compound **14**, clearly assigning five 3d electrons to each iron atom. In the β spin channel, the last available electron must be equally distributed among degenerated eff-AOs associated with the two symmetry-equivalent Fe centers, with an occupation of 0.318. Consequently, the forced split of the last electron leads to the Fe (+2.5)/Fe­(+2.5) mixed valence OS and an overall *R*(%) = 61.3 value.

So far, the oxidation state (OS) assignments obtained through EOS analysis without any external guidance are entirely consistent with those derived from the iBOS or BVS methods outlined in the IUPAC report, both of which rely on either idealized bond orders or reference structural data. Next, we examine a series of dihydrogen/hydride (**16**-**23**) MH_4_ systems with *I*_4_/*mmm* symmetry, consisting of 10 atoms per unit cell: four hydridic hydrogens (H_b_), two molecular hydrogens (H_a_-H_a_), and two metal cations (see Figure S7). These structures were reported by Bi and Zurek^26^ in their investigation of the superconducting properties of metal tetrahydrides, focusing on the roles of hydridic and molecular hydrogen atoms. To determine the OS, they relied on QTAIM charges and H_a_-H_a_ bond lengths, concluding that the H_b_ hydrogen atoms exhibit hydride-like behavior, as indicated by their calculated partial charges (ranging from −0.46 to −0.73) across all compounds. The QTAIM charge for the H_a_-H_a_ unit in divalent metal-containing compounds (M = Mg, Ca, Sr), was close to zero, suggesting a formally neutral H_2_ (0) character. However, in compounds with trivalent metal atoms (M = Sc, Y), the H_a_-H_a_ bonds were stretched and weakened, with partial charges significantly higher than those in divalent metal systems, which were interpreted as formally ionic H_2_ (1-) units. This effect was further amplified in compounds containing tetravalent metal atoms (M = Zr, Ce, Th), where the H_a_-H_a_ bonds elongate to over 1.3 Å, and the H_a_ QTAIM charges exceed −0.67. As a result, the authors considered both H_a_ and H_b_ centers as formally hydridic.

We have applied EOS analysis considering two different fragmentation patterns, namely atomic-wise and also considering the H_a_-H_a_ units as a fragment. The values of the reliability index *R*(%) can then be used as a guide to select the most appropriate picture.

The results of the EOS analysis for the compounds **16**-**18** involving alkaline earth metals with 12 valence electrons per unit cell are gathered in Table S7. For instance, in the MgH_4_ structure (**16**), when using atomic fragments one can see that the eff-AO occupations of centers H_a_ (0.467) and H_b_ (0.730) differ significantly. The highest occupied eff-AO on the Mg centers has an occupation of merely 0.08, pointing toward a clear Mg (2+) ion. In the EOS procedure, four α and four β electrons are assigned to the H_b_ centers, which become hydride, and the remaining two α and two β valence electrons must be distributed among the symmetry-equivalent H_a_ centers, for a formal *R*(%) = 50 value. When the H_a_-H_a_ unit is considered as a fragment in the EOS procedure the analysis becomes more straightforward. In this case, one α and one β electron are unambiguously assigned to each unit, which displays an EFO of 0.89. The situation is essentially the same for M = Ca and M = Sr., Going down the group, the only effect is a slight elongation of the H_a_-H_a_ bond, which directly translates in a slight increase in the occupation of the eff-AO for the H_a_ centers.

In the structures with M = Sc (**19**) and M = Y (**20**) there is one more α and one more β valence electrons to distribute. When using atomic fragmentations, the difference in the occupation of the eff-AOs for H_a_ and H_b_ is reduced compared to the previous example (see Table S8). In the case of ScH_4_ we obtain an occupation of 0.626 for H_b_ and 0.472 for H_a_. The former has more marked hydridic character and in fact the EOS procedure assigns one α (and one β) electron to H_b_, becoming H_b_(1-). The last three α and β electrons must be assigned to the four symmetry-equivalent Ha centers, because the occupation of the first eff-AO of Sc (0.186) is larger than for the previously discussed group II ions, but still too low compared to that of the H_a_ centers. On average, each H_a_ centers receives 3/4 + 3/4 electrons, resulting in a fractional H_a_(−1/2) OS because of symmetry. Considering the H_a_-H_a_ units as fragments results leads to somewhat worst description, because for each spin channel, after each unit receives one electron, there is still one last electron left to assign. The candidates at this point are the second EFO on each H_a_-H_a_ unit, with occupation of 0.192, and the aforementioned eff-AO on Sc, with an occupation of 0.186. Still, the EOS scheme assings an additional half-electron per spin channel to each H_a_-H_a_ unit, resulting in a formal (−1), but the assignment is much more ambiguous.

Finally, for M = Zr, Ce, or Th, there are 16 valence electrons per unit cell. The H_a_-H_a_ bond distance is quite large, particularly for the heaviest elements. Repeating the same process we observe now that higher *R*(%) values are obtained by considering each atom as a fragment, as compared to considering H_a_-H_a_ units (see Table S9). The occupations of the eff-AOs of H_b_ are still larger than for H_a_ in all cases, and the occupation of the first eff-AO on the metal M is quite significant, especially for M = Zr (0.247). Still, the eight valence electrons per spin channel are assigned to each of the four H_a_ and H_b_ centers, with an *R*(%) = 75.4 for the assignment. Considering H_a_-H_a_ units leads to the same OS assignment, with H_a_-H_a_ being formally dianionic (−2) because the occupation of their second EFO (0.366) is still larger than that of the metal. However, the reliability index of the assignment is clearly worse. The same picture is consistently obtained for the M = Ce and M = Th solids. As the occupation of the eff-AO of the metal decreases down the group, the values of *R*(%) correspondingly increase. Hence, the electronic structure is best described as M^4+^(H^–^)_4_ in all cases.

Under sufficiently high pressures, highly electronegative elements like fluorine and oxygen can oxidize xenon atoms, engaging the valence electrons of the noble gas to form stable compounds. Zhu et al.^27^ predicted the existence of thermodynamically stable Xe–O compounds at high pressures, with XeO, XeO_2_, and XeO_3_ becoming stable at pressures above 83, 102, and 114 GPa, respectively. The authors relied on structural parameters and the shape of the ELF to assign OS. Specifically, they claimed that the Xe atoms with fully filled electron configurations (Xe^0^ and Xe^6+^) would exhibit spherical ELF distribution, whereas those with unfilled shells (e.g., Xe^2+^ or Xe^4+^) would not.

The results of EOS analysis for all structures reported by Zhu et al.^27^ (**24–29**) are gathered in Tables S10–S12. At 100 GPa, the structure XeO (**24**) (see Figure S10) features a unit cell containing eight atoms. The four Xe atoms are symmetry-equivalent, with alternating Xe–O bond lengths of 2.0 Å and 2.1 Å. According to the EOS analysis, two of the three Xe 5p eff-AOs are occupied with an occupation of 0.796. At the same time, the third Xe 5p eff-AO has a reduced occupation of 0.444, making it formally unoccupied and serving as the frontier eff-AO. This leads to a quite clear formal Xe­(2+) O­(2-) picture, with *R*(%)= 74.6. The original study reached the same conclusion based on the presence of a toroidal maximum in the ELF around each Xe atom.

At 200 GPa, the resulting structure (**25**) exhibits significant differences. It comprises two distinct types of xenon atoms with differing environments. Xe2 remains unbonded to any atoms, whereas Xe1 is coordinated in a square planar arrangement, bonded to four oxygen atoms with bond lengths of 2.0 Å and 2.1 Å. Assuming an OS of −2 for each oxygen atom, the original study characterized the Xe1 atoms as (+4), forming chains akin to XeO_2_, and formally neutral Xe2 centers. This OS picture is also supported by EOS analysis. In the Xe2 atom, the three 5p-type eff-AOs are considered occupied and are quasi-degenerated (0.78–0.74), indicating an isotropic environment. However, in the Xe1 atom, only the first 5p eff-AO is formally occupied. The occupation of the first unoccupied eff-AO on Xe1 is significant (0.477), but clearly smaller than the last occupied on the oxygen centers (0.709). The electron sharing between these centers is substantial, leading to QTAIM charges strongly deviating from the ideal values (e.g., + 1.71 for Xe1 and ca. −1.0 for O1 and O2). Still, EOS analysis leads to a quite clear-cut Xe2(0)­Xe1­(+2)­O(−2)_2_ OS assignment, with *R*(%) = 73.3. Notably, when the XeO_2_ (Xe1O1O2) chain is treated as a fragment, its formal charge is calculated to be 0, and the Xe2 atom also exhibits an OS of 0, with a somewhat higher reliability index.

There are two different structures with XeO_2_ stoichiometry. The first one obtained at 150 GPa (**26**) has the xenon atoms (Xe1 and Xe2) in a slightly nonplanar square coordination with four Xe–O bonds. The QTAIM partial charges are very close to +2 for all Xe and −1 for all O centers. Hence, Zhu et al.^27^ relied on the shape of the ELF around the Xe nuclei for the OS assignment. They found two maxima of the ELF, which can be put into correspondence with two lone pairs, and hence an indicator of Xe (+4) ions. EOS analysis shows a large degree of electron sharing in the XeO_2_ units. Each Xe has two eff-AOs formally occupied, which rather than two lone pairs should correspond to a 5s and a 5p-type eff-AOs. The respective occupations are 0.929 and 0.784, and 0.925 and 0.775 for Xe1 and Xe2, respectively. The resulting OS assignment is a very clear Xe­(+4) O(−2)_2_ with *R*(%) = 77.6.

The second structure at 200 GPa (**27**) is different because it can be represented as having parallel xenon chains (Xe–Xe bonds distance of 2.62Å) and each Xe atom has two bonds with oxygen atoms (see Figure S11). The authors did not attempt an OS assignment for this structure, possibly because again the QTAIM charges are very far from the ideal values already for the oxygen centers and the ELF did not provide enough clues. The results of EOS analysis, on the other hand, are not too different from the other structures studied. The occupation of the 5p eff-AOs of the Xe centers are 0.605, 0.518, and 0.367. One could be tempted to consider two 5p-type eff-AOs as occupied but again, only one of the results formally occupied upon the EOS procedure. This is because the occupation of the fourth eff-AO on the oxygen centers (0.731) is still larger. This emphasizes again the fact that eff-AO occupations must not be rounded to the nearest integer to infer OS. The final picture is Xe­(+4) O(−2)_2_ with a somewhat smaller *R*(%) = 58.7 value due to competing eff-AOs on the Xe centers.

There are two additional structures with stoichiometry XeO_3_, namely **28** at 130 GPa and **29** at 150 GPa. Structure **28** is shown in Figure S12, and it can be seen as composed of two sublattices: square XeO_2_ (XeO1O1) chains and linear chains made of O_2_ (O2–O2) dumbbells. Zhu et al.^27^ relied upon the shape of the ELF around the Xe atoms to determine their OS, assigning it as Xe­(+4) and assumed formal oxo O1­(2-) and embedded neutral O_2_ units. The results of the EOS analysis are gathered in Table S12. The QTAIM charges of Xe, O1, and O2 are +2.30, −0.89, and −0.52, respectively. The partial charge on Xe is notably higher than that observed in XeO or XeO_2_ solids, while the O_2_ (O2–O2) unit is electron-rich, with a partial charge of −1.04. This electron distribution complicates the straightforward assignment of OS.

When using atomic fragments in the EOS procedure, the analysis suggests an Xe­(+4) O1(−2) O2(0) configuration. The reliability index *R*(%) = 60.9 is rather low, because of the significant occupation of the first unoccupied eff-AO on the O2 centers. However, when the O2 (O2–O2) units are treated as a single fragment in the EOS analysis, a different OS assignment emerges. As shown in Table S12, the electron-rich O_2_ units are formally assigned as O_2_(2-), and the last electrons (α and β) are equally distributed between two symmetry-equivalent Xe centers, resulting in a mixed-valence Xe­(+5) picture. Despite this alternative assignment, the reliability index for this configuration is lower than that obtained using atomic fragments, which prevails as the preferred and more consistent OS assignment for this system.

The situation in (**29**) is even more cumbersome. There are two types of Xe centers and five types of O centers in the unit cell. The QTAIM charges of Xe1 and Xe2 are +2.47 and +2.43. Among the different oxygen centers, O1 has a charge of −0.55 and the remaining O centers have a similar charge of ca. −0.90.

The reliability of the assignment according to EOS is very low, with *R*(%) = 51.2. The main reason is not on the different oxygen atoms but on the two Xe centers. Despite having very similar partial charge, the EOS procedure only assigns one electron pair to the Xe1 centers, becoming formally Xe­(6+). The last electrons are assigned to Xe2 centers, becoming formally Xe­(4+). The occupations of the last occupied eff-AO on Xe2 (0.540) and the first unoccupied eff-AO on Xe1(0.528) are mince, hence the overall *R*(%) = 51.2 value. In addition, the O1 centers are again electron-rich, so they are pictured as O1(0) but with an available eff-AO with occupation 0.513. All in all, the OS assignment in this structure is intrinsically ambiguous. In the original paper, we could not find detailed information about this structure to confront our assignment. Nevertheless, EOS analysis does identify xenon atoms with significant Xe­(+6) character in high-pressure XeO_3_ solids.

Xenon atoms can also be stabilized in compounds as fluorides, namely XeF_2_ (**30**), XeF_3_ (**31**), and XeF_4_ (**32**). The results of EOS analysis for these systems are summarized in Table S13. The reliability of the OS assignments is very high in all cases, with *R*(%) values between 89 and 98. In the linear XeF_2_ (**30**), the Xe atoms are oxidized to the +2 OS forming covalent bonds with F by sharing its 5p electrons. The phase XeF_3_ (**31**)^36^ essentially consists of a mixture of discrete XeF_2_ (Xe1, F1, F1) and XeF_4_ (Xe2, F2, F2, F3, F3) molecules. The two chemically different kinds of xenon atoms are fully established in our analysis where the linear coordinated Xe1 atoms are described better as Xe­(+2) and Xe2 atoms with square-planar configurations have an OS of +4. Finally, the XeF_4_ structure^37^ can be characterized as square planar molecules of XeF_4_ with F–Xe–F bond angles of 89.7° and 90.3°. EOS analysis clearly points toward Xe­(4+) formal ions.

On the other hand, the possibility of xenon atoms gaining electrons and becoming formally anionic has also been explored. Miao and co-workers^28^ explored the formation of Mg–Xe compounds under high-pressure conditions. Their studies revealed that MgXe crystallizes as an alternative stacking of Mg and Xe square lattices at the center sites with space group *Pm*-3*m* at pressures exceeding 100 GPa. The QTAIM charge on the Xe atoms was as large as −1.40, suggesting a pronounced anionic character for xenon in this compound.

We have applied EOS analysis to (**33**) to confirm the formal anionic nature of the Xe centers. They are in a perfect octahedral environment, which is reflected in the occupation of the eff-AOs. We obtain eff-AOs with occupation (degeneracy) 0.958 (1), 0.843(3), 0.211 (3), and 0.112 (2), associated with the 5s, 5p and 5d shells, respectively. The first eff-AO on the Mg centers has an occupation of 0.115. The five α (and five β) electrons are thus assigned to the Xe centers, for a formal Xe­(2-) Mg­(2+) picture. The reliability index is very low (*R*(%) = 59.6) because, by symmetry constraints, the excess charge on Xe is almost evenly distributed among the 5d shell.

High pressures also can activate core electrons. This is the case of the HgF_4_ compound reported by Botana et al,.^29^ where the oxidation state (OS) of mercury is predicted to shift from the typical +2 to an unusual +4, involving the activation of its semicore 5d electrons. This conclusion was originally drawn from the analysis of the projected density of states (PDOS), which revealed a splitting of the 5d band between the valence and conduction bands. Herein, we analyze the compounds (**34–36**) obtained at 50, 100, and 200 GPa. The three structures exhibit the same symmetry (*I*4/*m*) and bonding patterns. The semicore and valence shell of Hg (5d^10^6s^2^) consists of 12 electrons, and according to EOS analysis only four eff-AOs are considered doubly occupied. The first unoccupied eff-AO on Hg has a quite large occupation (0.684 in structure **34**), consistent with the relatively low QTAIM charge of +1.76 on Hg. This occupation competes with that of the last occupied eff-AO on the fluorine centers (0.747). As a result, the reliability of the OS assignment is relatively low, with *R*(%) = 56.3. As pressure increases, both the QTAIM charge on Hg and the *R*(%) values show a monotonic rise, making the Hg­(+4) picture somewhat more plausible. This trend suggests that higher pressures enhance the likelihood of mercury adopting a + 4 oxidation state, although the reliability of this assignment remains moderate.

Finally, to explore the limits of applicability of the EOS scheme, we have also examined solids featuring homonuclear bonds. These systems present one of the most challenging scenarios for oxidation state (OS) assignment due to the minimal polarity of such bonds. According to IUPAC guidelines, the ionic approximation for homonuclear bonds should be approached homolytically. However, the EOS scheme does not make any specific distinctions or provisions for this situation.

The set of compounds is displayed in Figure S16 and the results of EOS analysis are summarized in Table S16. The CsH_3_ polyhydride (**37**) and the CsF_3_ multifluoride^32^ (**38**) feature symmetric linear H_3_ and F_3_ units, respectively. EOS analysis considering the entire H_3_ and F_3_ units as fragments results in a rather trivial and unambiguous OS assignment, with formal anionic triatomic linear chains. When considering separate atomic centers the reliability of the OS assignment naturally decreases, because of the formal splitting of homonuclear bonds. Still in both the formal H_3_ (−1) and F_3_ (−1) units the central atom is best described as formally cationic (+1), so that the ionic approximation of the homonuclear bonds goes in the direction of the external atoms. This picture is analogous to that obtained for the isolated triiodide anion.^4^ The *R*(%) values drop to 68 and 66 using atomic fragments, but EOS analysis finds the difference between the different H and F atoms still significant.

At zero pressure, CsF_5_ (**39**) shows a stable *P*2_1_ phase with five-member V-shaped F chains, and F–F bond lengths varying from 1.6 to 1.9 Å. EOS analysis considering the whole F_5_ unit results in a trivial Cs­(+1)­F_5_(−1) assignment. Using atomic fragments the central F1 center is clearly identified as F(−1), whereas the other F2 and F3 centers strongly compete for the electrons with a small difference in the occupation of the frontier eff-AOs (0.589 vs 0.524). Thus, this structure can also be characterized as Cs­(1+) F­(1-) F_2_(0).

The compound (**40**) described by Peng et al.^31^ features Li^+^ and pentazolate (N_5_^–^) ions. In an earlier work,^4^ we considered pentazole molecule N_5_H, for which EOS analysis breaks down due to the extremely unpolarized N–N bonds. The same conclusion can be drawn from the analysis of (**40**) shown in Table S16. The occupation of the valence eff-AOs of the different N atoms of the ring (all five atoms are inequivalent in the unit cell) is very similar, leading to a completely ambiguous assignment with *R*(%) = 50.

## Conclusions

We have successfully implemented the generalization of the EOS analysis to solid-state calculations. Technically, the algorithm is realized from the atomic overlap matrices (AOM) of the atoms of the unit cell, determined within the framework of QTAIM. If the solid is an insulator, the AOMs benefit from the transformation to the maximally localized Wannier functions, as performed by the *critic2* code. Otherwise, the AOMs are obtained directly in the basis of Bloch states.

The main ingredient of EOS analysis is the effective atomic orbitals (eff-AOs) and their occupations, easily obtained from the eigenvectors and eigenvalues of the respective AOMs. Once they are obtained for all atoms or fragments within the unit cell, the EOS algorithm proceeds in essentially the same fashion as in the molecular case. The scheme is readily extendable to any real-space partitioning, including Voronoi polyhedral or Hirshfeld-type partitionings, if the respective AOMs are available.

As a first test, we have considered all the solid-state structures discussed in the comprehensive IUPAC technical report of Karen et al.^3^ The OS assignments derived from the EOS analysis are fully consistent with those predicted by two established methods: the ionized bond order sums (which assumes idealized bond orders) and the bond valence sum (which relies on geometric reference data). The EOS scheme is broadly applicable without the need for external provisions or reference data, and it possesses the ability to self-assess its limits of applicability. This is achieved through the reliability index, which quantifies the deviation of the electronic structure from the ionic model for all atoms within the unit cell. This feature allows the method to identify cases where its predictions may be less reliable, providing a built-in measure of uncertainty/ambiguity in the OS assignment.

The EOS analysis has been subjected to a more rigorous evaluation by applying it to high-pressure systems exhibiting unconventional bonding patterns and OS. The OS assignments derived from the algorithm underline the fact that partial atomic charges (QTAIM in the present case) should never be used to infer oxidation states. Additionally, the method has been tested on electrides, where accurately determining the topology of the electron density—specifically, identifying the locations of non-nuclear attractors—is a critical prerequisite.

All in all, EOS scheme generalized to solids represents a new robust tool for the rational assignment of oxidation states from wave function analysis in the condensed phase.

## Computational Details

All calculations were carried out with Quantum ESPRESSO^38^ software and the PBEsol density functional.^39^ To yield the accurate QTAIM atomic basis an all-electron density calculation is required. For this reason, in this work, the all-electron densities were reconstructed from an additional PAW calculation^40^ at the same geometry and using the same grid. The electron density and Bloch states were obtained using a norm-conserving pseudopotential calculation. For the solids compound studied here, a cutoff of 80 Ry for the wave functions, 320 Ry for the density, and a 4 × 4 × 4 *k*-point grid was used. MLWFs were obtained using the wannier90 code.^41^ Atomic overlap matrices were obtained with the critic2 ^42–44^ code based on MLWFs and using the grid-specific integration method of Henkelman et al.^45–47^ Compounds with partially filled bands (metals) can not be calculated with MLWFs at present due to the ill-defined Wannier transformation. Instead, the Bloch states resulting from the SCF calculation are used directly. Spin-resolved effective fragment orbitals and subsequent effective oxidation state (EOS) analysis were performed with the APOST-3D program.^48^

## Supplementary Material



## Notes

Tables (S1–S16) gathering the occupation of frontier eff-AOs and the OS assignations for solids **1-40**. Figures (S1–S16) showing the atoms in the unit cell and their numbering for solids **1–40** (PDF)
