# Supplementary material for: Effective Oxidation State Analysis for Solids
Source: J Chem Theory Comput. 2025 Jul 3;21(14):7075–86. doi: 10.1021/acs.jctc.5c00482 (PMC12287994; doi:10.1021/acs.jctc.5c00482)
Supplement: Supplementary file 1 [file ct5c00482_si_001.pdf]

Supporting Information

Effective Oxidation States Analysis for Solids

Gerard Comas-Vilà,[1] Leila Pujal [1,2] Alberto Otero-de-la-Roza,[3] Davide Tiana,[4] Julia Contreras-Garcia [5,] and Pedro Salvador[1,]**

Table S1: Results of the EOS analysis for CrO_3. Assigned OS, occupation of the frontier eff-AOs ($\lambda_{LO}/\lambda_{FU}$) for each center (overall frontier eff-AOs marked in bold), and reliability index R(%) of the assignation.

ID Molecule	Atom/Fragment	EOS	$\lambda_{LO}/\lambda_{FU}$	Partial Charge	R(%)	Reference OS
**(1) CrO_3**	Cr	**+6**	core/**0.431**	+1.76	71.12	+6
	O1	**-2**	0.717/0.015	-0.84		-2
	O2	**-2**	0.645/0.023	-0.47		-2
	O3	**-2**	**0.643**/0.023	-0.46		-2

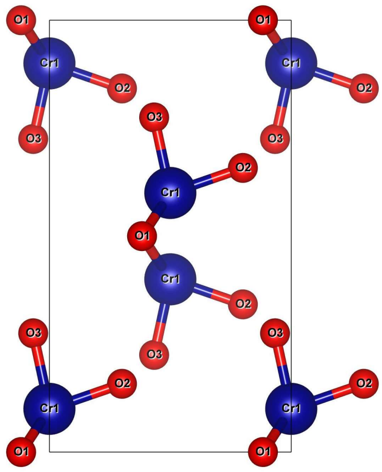

CrO_3, **1**

Figure S1: Structures and numbering of the case study from Table S2.

Table S2: Results of the EOS analysis for perovskites compounds. . Assigned OS, occupation of the frontier eff-AOs ($\lambda_{LO}/\lambda_{FU}$) for each center (overall frontier eff-AOs marked in bold), and reliability index *R*(%) of the assignation.

ID Molecule	Atom/Fragment	EOS	$\lambda_{LO}/\lambda_{FU}$	*R*(%)	Reference OS
**(2) $KMgF_3$**	K	**+1**	core/0.023	100	+1
	Mg	**+2**	core/**0.051**		+2
	F	**-1**	**0.939**/0.013		-1
**(3) $NaBF_4$**	Na	**+1**	core/0.021	100	+1
	B	**+3**	core/**0.086**		+3
	F1	**-1**	**0.899**/0.024		-1
	F2	**-1**	0.899/0.023		-1
**(4) $SrBe(OH)_4$**	Sr1	**+2**	core/**0.050**	100	+2
	Sr2	**+2**	core/0.046		+2
	Be	**+2**	core/0.047		+2
	OH (O1H1)	**-1**	**0.921**/0.020		-1
	OH (O4H2)	**-1**	0.922/0.020		-1

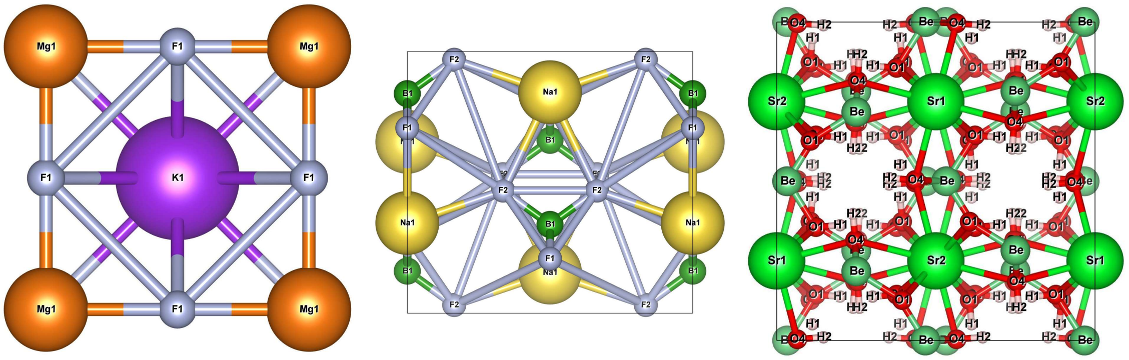

$KMgF_3$, **2** $NaBF_4$, **3** $SrBe(OH)_4$, **4**

Figure S2: Structures and numbering of the case studies from Table S3.

Table S3: Results of the EOS analysis for inverse perovskites compounds and a potassium electride. Assigned OS, occupation of the frontier eff-AOs ($\lambda_{LO}/\lambda_{FU}$) for each center (overall frontier eff-AOs marked in bold), and reliability index *R*(%) of the assignation.

ID Molecule	Atom/Fragment	EOS	$\lambda_{LO}/\lambda_{FU}$	*R*(%)	Reference OS
**(5) Cs_3AuO**	Cs	**+1**	core/**0.088**	100	+1
	Au	**-1**	0.873/0.056		**-1**
	O	**-2**	**0.802**/0.041		**-2**
**(6) Rb_3AuO**	Rb	**+1**	core/**0.063**	100	+1
	Au	**-1**	0.867/0.018		**-1**
	O	**-2**	**0.832**/0.009		**-2**
**(7) Ca_3AuN**	Ca	**+2**	core/**0.144**	60.4	+2
	Au	**-3**	**0.248**/0.230		**-3 or -1**
	N	**-3**	0.783/0.051		**-3**
**(8) K**	K1	**+1**	core/**0.127**	63.2	
	K2	**+1**	core/0.084		
	NNA	**-2**	**0.259**/0.005		

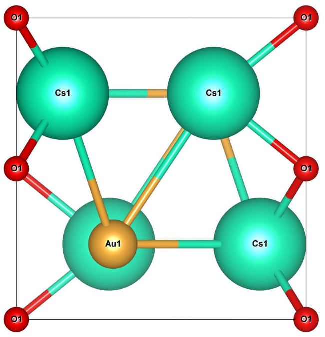

Cs_3AuO, **5**

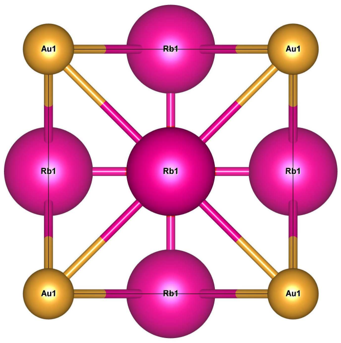

Rb_3AuO, **6**

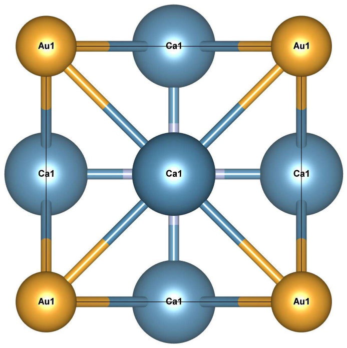

Ca_3AuN, **7**

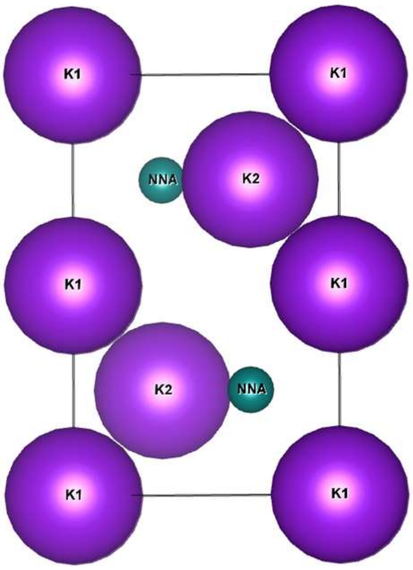

K, **8**

Figure S3: Structures and numbering of the case studies from Table S4

Table S4: Results of the EOS analysis for GaSe. Assigned OS, occupation of the frontier eff-AOs ($\lambda_{LO}/\lambda_{FU}$) for each center (overall frontier eff-AOs marked in bold), and reliability index *R*(%) of the assignation.

ID Molecule	Atom/Fragment	EOS	$\lambda_{LO}/\lambda_{FU}$	*R*(%)	Reference OS
**(9) GaSe**	Ga-Ga	**+4**	0.916/**0.429**	77.7	+4
	Se	**-2**	**0.706**/0.023		-2
	Ga	**+2**	**0.581**[a]/**0.217**	50.0	+2
	Se	**-2**	0.706/0.023		-2

[a] Formal split of the last electron pair among two identical Ga centers.

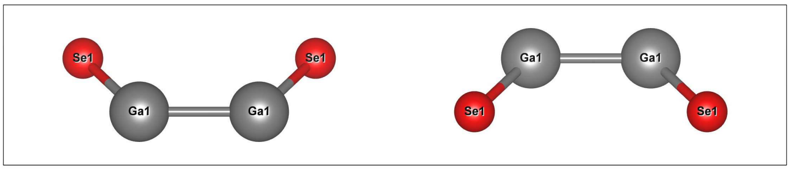

GaSe, **9**

Figure S4: Structures and numbering of the case study from Table S5.

Table S5: Results of the EOS analysis for Ba_3Si_4. Assigned OS, occupation of the frontier eff-AOs ($\lambda_{LO}/\lambda_{FU}$) for each center (overall frontier eff-AOs marked in bold), and reliability index *R*(%) of the assignation.

ID Molecule	Atom/Fragment	EOS	$\lambda_{LO}/\lambda_{FU}$	*R*(%)	Reference OS
**(10) Ba_3Si_4**	Ba1	**+2**	core/**0.136**	100	+2
	Ba2	**+2**	core/0.118		+2
	Ba3	**+2**	core/0.121		+2
	Si_4	**-6**	**0.717**/0.049		-6
	Ba1	**+2**	core/**0.136**	51.7	+2
	Ba2	**+2**	core/0.118		+2
	Ba3	**+2**	core/0.121		+2
	Si1	**-2**	0.449/**0.404**		
	Si2	**-1**	***0.421***/0.400		

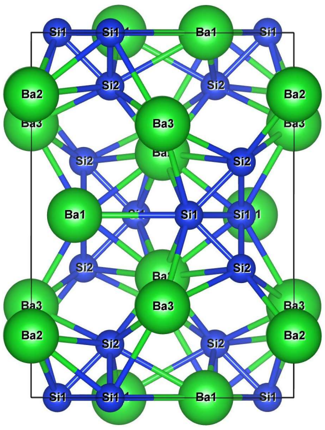

Ba_3Si_4, **10**

Figure S5: Structures and numbering of the case study from Table S6.

Table S6: Results of the EOS analysis for transition metal compounds. Assigned OS, occupation of the frontier eff-AOs ($\lambda_{LO}/\lambda_{FU}$) for each center (overall frontier eff-AOs marked in bold), and reliability index *R*(%) of the assignation.

ID Molecule	Atom/Fragment	EOS	$\lambda^{\alpha}_{LO}/\lambda^{\alpha}_{FU}$	$\lambda^{\beta}_{LO}/\lambda^{\beta}_{FU}$	*R*(%)	Reference OS
**(11) Cs_2Pt**	Cs1	+1	core/0.069		100	+1
	Cs2	+1	core/**0.078**			+1
	Pt	-2	**0.783**/0.009			-2
**(12) WCl_4**	W	+4	0.995/**0.351**		92.6	+4
	Cl1	-1	0.812/0.022			-1
	Cl2	-1	**0.777**/0.020			-1
	Cl3	-1	0.841/0.020			-1
**(13) Rb_2CuCl_4**	Cu	+2	0.963/**0.202**	0.917/**0.496**	75.4	+2
	Rb	+1	core/0.029	core/0.028		+1
	Cl1	-1	**0.856**/0.028	**0.750**/0.027		-1
	Cl2	-1	0.877/0.020	0.781/0.018		-1
**(14) $YBaFe_2O_5$**	Ba	+2	core/0.054	core/0.056	55.6	+2
	Y	+3	core/0.097	core/0.092		+3
	Fe1	+3	0.939/0.167	core/**0.282**		+3
	Fe2	+2	0.941/**0.173**	**0.338**/0.266		+2
	O1	-2	0.832/0.024	0.741/0.017		-2
	O2	-2	**0.825**/0.028	0.763/0.021		-2
	O3	-2	0.838/0.031	0.747/0.020		-2
	O4	-2	0.838/0.031	0.761/0.022		-2
**(15) $YBaFe_2O_5$**	Ba	+2	core/0.052	core/0.055	61.3	+2
	Y	+3	core/0.091	core/0.088		+3
	Fe	+2.5	0.944/**0.169**	***0.318***[a]/**0.205**		+2.5
	O1	-2	**0.834**/0.030	0.741/0.015		-2
	O2	-2	0.835/0.030	0.758/0.023		-2
	O3	-2	0.835/0.030	0.758/0.023		-2

[a] Formal split of the last electron among two identical Fe centers.

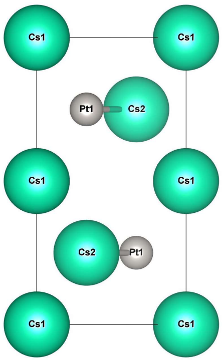

Cs_2Pt, **11**

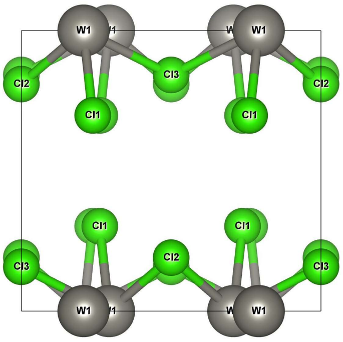

WCl_4, **12**

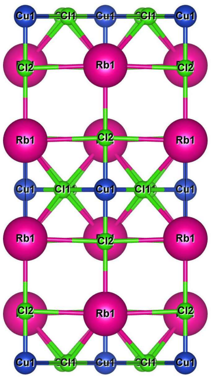

Rb_2CuCl_4, **13**

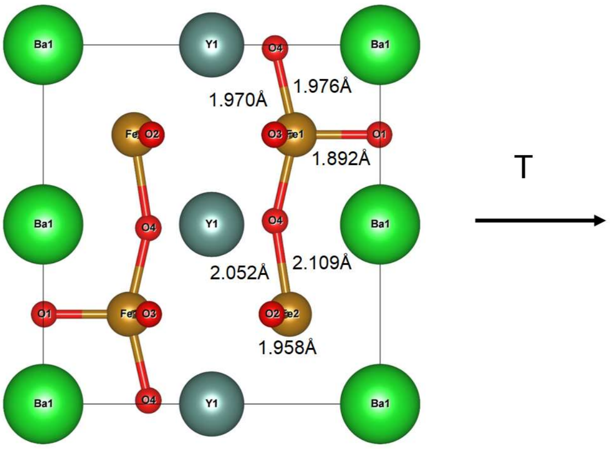

$Ba_2YFe_2O_5$, **14**

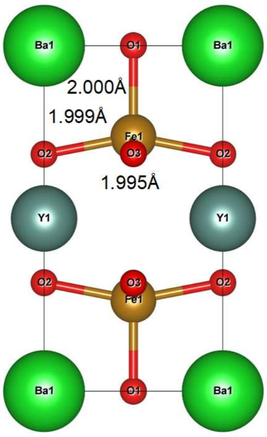

$Ba_2YFe_2O_5$, **15**

Figure S6: Structures and numbering of the case studies from Table S7.

Table S7: Results of the EOS analysis for hydridic and molecular hydrogen atoms in divalent metal complexes. Assigned OS, occupation of the frontier eff-AOs ($\lambda_{LO}/\lambda_{FU}$) for each center (overall frontier eff-AOs marked in bold), and reliability index R(%) of the assignation.

ID Molecule	Atom/Fragment	EOS	$\lambda_{LO}/\lambda_{FU}$	Partial Charge	R(%)	H-H distance (Å)	Reference OS
(16) MgH_4	H_a-H_a	**0**	0.890/0.078	-0.213		0.757	**0**
	H_b	**-1**	**0.730**/0.032	-0.717	**100**		**-1**
	Mg	**+2**	core/**0.080**	1.650			**+2**
	H_a	**0**	***0.467***[a]/0.025	-0.106			
	H_b	**-1**	0.730/0.032	-0.717	50		
	Mg	**+2**	core/**0.080**	1.650			
(17) CaH_4	H_a-H_a	**0**	0.911/0.061	-0.155		0.767	**0**
	H_b	**-1**	**0.773**/0.019	-0.703	**100**		**-1**
	Ca	**+2**	core/**0.072**	1.558			**+2**
	H_a	**0**	***0.473***[a]/0.019	-0.077			
	H_b	**-1**	0.773/0.019	-0.703	50		
	Mg	**+2**	core/**0.072**	1.558			
(18) SrH_4	H_a-H_a	**0**	0.912/0.047	-0.076		0.765	**0**
	H_b	**-1**	**0.749**/0.015	-0.622	**100**		**-1**
	Sr	**+2**	core/**0.069**	1.324			**+2**
	H_a	**0**	***0.469***[a]/0.014	-0.038			
	H_b	**-1**	0.749/0.015	-0.622	50		
	Sr	**+2**	core/**0.069**	1.324			

[a] Formal split of the last electron pair among two identical H_a centers.

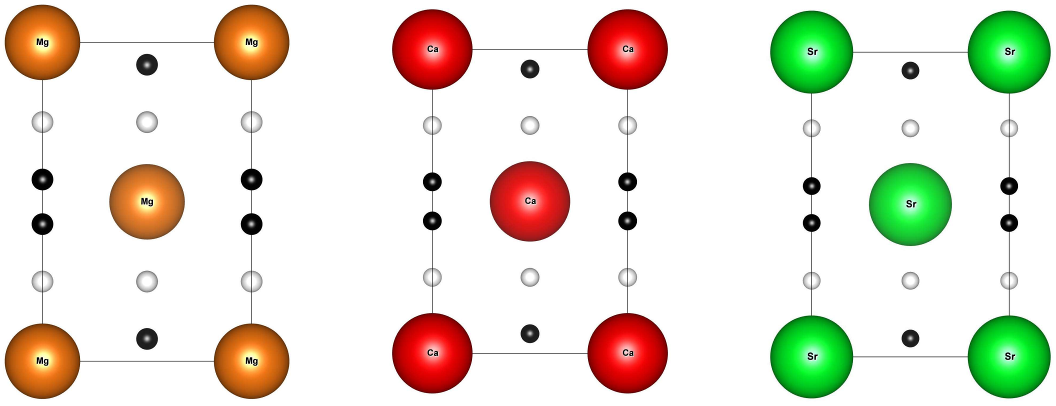

MgH_4, **16** CaH_4, **17** SrH_4, **18**

Figure S7: Structures and numbering of the case studies from Table S8. H_a atoms in black and H_b atoms in white spheres.

Table S8: Results of the EOS analysis for hydridic and molecular hydrogen atoms in trivalent metal complexes. Assigned OS, occupation of the frontier eff-AOs ($\lambda_{LO}/\lambda_{FU}$) for each center (overall frontier eff-AOs marked in bold), and reliability index *R*(%) of the assignation.

ID Molecule	Atom/Fragment	EOS	$\lambda_{LO}/\lambda_{FU}$	Partial Charge	R(%)	H-H distance (Å)	Reference OS
	H_a-H_a	**-1**	***0.192***[a]/0.048	-0.329		0.867	**-1**
	H_b	**-1**	0.626/0.034	-0.508	50		**-1**
**(19) ScH_4**	Sc	**+3**	core/**0.182**	1.349			+3
	H_a	**-1/2**	***0.472***[a]/0.036	-0.165			
	H_b	**-1**	0.626/0.034	-0.508	50		
	Sc	**+3**	core/**0.182**	1.349			
	H_a-H_a	**-1**	***0.196***[a]/0.043	-0.371		0.911	**-1**
	H_b	**-1**	0.661/0.033	-0.562	50		**-1**
**(20) YH_4**	Sc	**+3**	core/**0.159**	1.499			+3
	H_a	**-1/2**	***0.489***[a]/0.038	-0.186			
	H_b	**-1**	0.661/0.033	-0.562	50		
	Sc	**+3**	core/**0.159**	1.499			

[a] Formal split of the last electron pair among two identical fragments

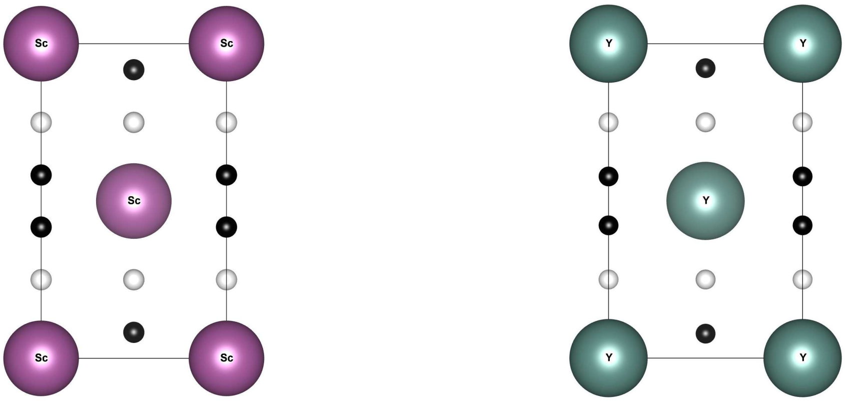

ScH_4, **19** YH_4, **20**

Figure S8 : Structures and numbering of the case studies from Table S9. Black balls represent H_a-H_a units and white balls represent H_b units.

Table S9: Results of the EOS analysis for hydridic and molecular hydrogen atoms in tetravalent metal complexesAssigned OS, occupation of the frontier eff-AOs ($\lambda_{LO}/\lambda_{FU}$) for each center (overall frontier eff-AOs marked in bold), and reliability index R(%) of the assignation.

ID Molecule	Atom/Fragment	EOS	$\lambda_{LO}/\lambda_{FU}$	Partial Charge	R(%)	H-H distance (Å)	Reference OS
**(21) ZrH_4**	H_a-H_a	**-2**	**0.366**/0.066	-0.563		1.331	-2
	H_b	**-1**	0.571/0.044	-0.441	61.9		-1
	Zr	+4	core/**0.247**	1.450			+4
	H_a	**-1**	**0.502**/0.043	-0.281			
	H_b	**-1**	0.571/0.044	-0.441	**75.4**		
	Zr	+4	core/**0.247**	1.450			
**(22) CeH_4**	H_a-H_a	**-2**	**0.417**/0.037	-0.563		1.566	-2
	H_b	**-1**	0.588/0.029	-0.382	75.2		-1
	Ce	+4	core/**0.166**	1.330			+4
	H_a	**-1**	**0.536**/0.029	-0.282			
	H_b	**-1**	0.588/0.029	-0.382	**86.4**		
	Ce	+4	core/**0.166**	1.330			
**(23) ThH_4**	H_a-H_a	**-2**	**0.491**/0.041	-0.729		1.664	-2
	H_b	**-1**	0.595/0.033	-0.417	80.8		-1
	Th	+4	core/**0.182**	1.546			+4
	H_a	**-1**	**0.567**/0.031	-0.365			
	H_b	**-1**	0.595/0.033	-0.417	**88.2**		
	Th	+4	core/**0.182**	1.546			

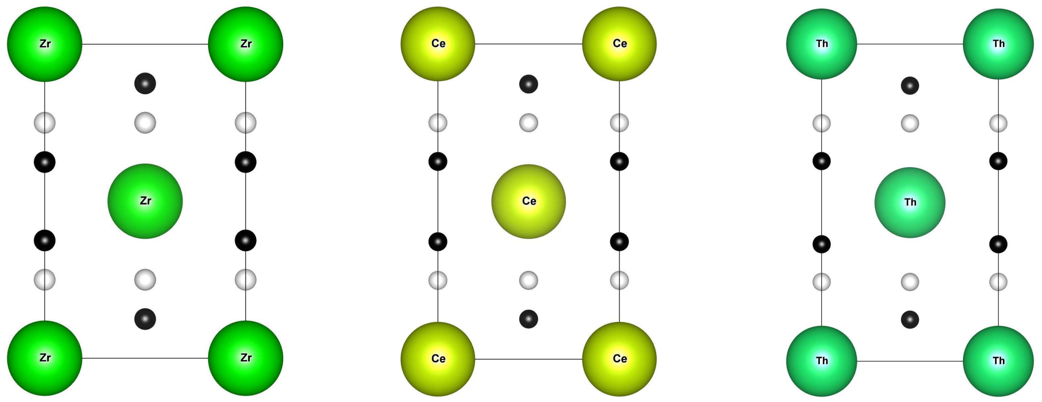

ZrH_4, **21** CeH_4, **22** ThH_4, **23**

Figure S9: Structures and numbering of the case studies from Table S10. Black balls represent H_a-H_a units and white balls represent H_b units.

Table S10: Results of the EOS analysis for xenon oxides with stoichiometry XeO. Assigned OS, occupation of the frontier eff-AOs ($\lambda_{LO}/\lambda_{FU}$) for each center (overall frontier eff-AOs marked in bold), and reliability index *R*(%) of the assignation.

ID Molecule	Pressure (GPa)	Space Group	Atom/Fragment	EOS	$\lambda_{LO}/\lambda_{FU}$	Partial Charge	*R*(%)	Reference OS
**(24) XeO**	100	*Pbcm*	Xe	**+2**	0.796/**0.444**	+0.99	74.6	+2
			O	**-2**	**0.691**/0.043	-0.99		-2
**(25) XeO**	200	P2$_1$/*m*	Xe1	**+4**	0.779/**0.477**	+1.71	73.3	+4
			Xe2	**0**	0.738/0.065	+0.44		0
			O1	**-2**	0.734/0.042	-1.06		-2
			O2	**-2**	**0.709**/0.046	-1.08		-2
			XeO_2 (Xe1O1O2)	**0**	0.746/**0.395**		**84.2**	
			Xe2	**0**	**0.738**/0.065			

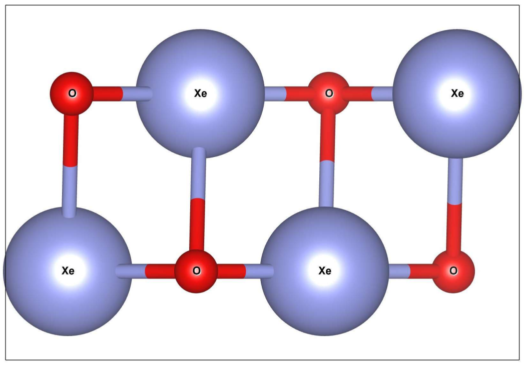

XeO, **24**

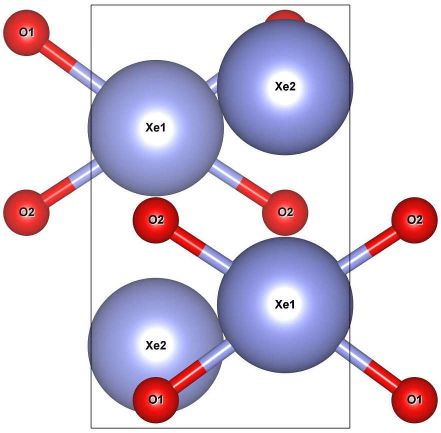

XeO, **25**

Figure S10: Structures and numbering of the case studies from Table S11.

Table S11: Results of the EOS analysis for xenon oxides with stoichiometry XeO_2. Assigned OS, occupation of the frontier eff-AOs ($\lambda_{LO}/\lambda_{FU}$) for each center (overall frontier eff-AOs marked in bold), and reliability index *R*(%) of the assignation.

ID Molecule	Pressure (GPa)	Space Group	Atom/Fragment	EOS	$\lambda_{LO}/\lambda_{FU}$	Partial Charge	*R*(%)	Reference OS
**(26) XeO_2**	150	$P2_1/c$	Xe1	**+4**	0.784/**0.432**	+1.97	77.6	+4
			Xe2	**+4**	0.775/0.410	+1.98		+4
			O1	**-2**	0.710/0.039	-0.98		-2
			O2	**-2**	0.725/0.039	-1.01		-2
			O3	**-2**	**0.709**/0.037	-0.99		-2
			O4	**-2**	0.717/0.039	-0.98		-2
**(27) XeO_2**	200	*Cmcm*	Xe	**+4**	**0.605/0.518**	+2.06	58.7	
			O	**-2**	0.731/0.035	-1.03		

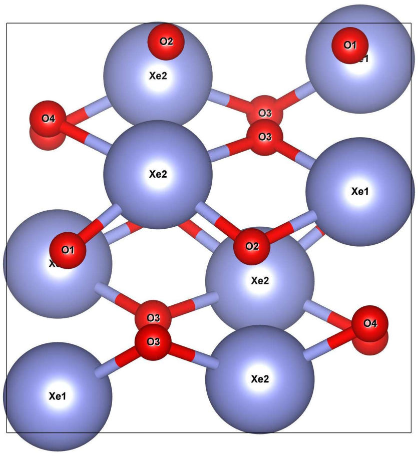

XeO_2, **26**

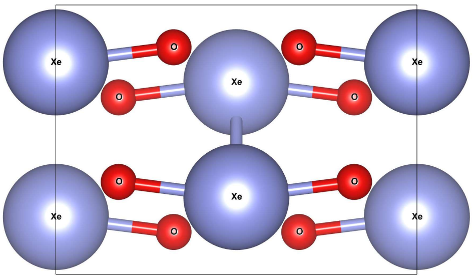

XeO_2, **27**

Figure S11: Structures and numbering of the case studies from Table S12.

Table S12: Results of the EOS analysis for Xenon oxides with stoichiometry XeO_3. Assigned OS, occupation of the frontier eff-AOs ($\lambda_{LO}/\lambda_{FU}$) for each center (overall frontier eff-AOs marked in bold), and reliability index *R*(%) of the assignation.

ID Molecule	Pressure (GPa)	Space Group	Atom/Fragment	EOS	$\lambda_{LO}/\lambda_{FU}$	Partial Charge	***R***(%)	Reference OS
**(28) XeO_3**	130	*$P4_2/mnm$*	Xe	**+4**	**0.606**/0.454	+2.30	60.9	+4
			O1	**-2**	0.731/0.035	-0.89		-2
			O2	**0**	0.771/**0.496**	-0.52		-2
			Xe	**+5**	0.755/<u>**0.606**</u> [a]	+2.30	50	
			O1	**-2**	0.731/0.035	-0.89		
			O_2 (O2O2)	**-2**	**0.697**/0.067	-1.04		
**(29) XeO_3**	150	*C*2/*c*	Xe1	**+6**	0.920/**0.528**	+2.47	51.2	
			Xe2	**+4**	**0.540**/0.441	+2.43		
			O1	**0**	0.781/0.513	-0.55		
			O2	**-2**	0.732/0.035	-0.90		
			O3	**-2**	0.729/0.035	-0.89		
			O4	**-2**	0.734/0.035	-0.89		
			O5	**-2**	0.743/0.037	-0.86		

[a] Formal split of the last electron pair among two identical fragments

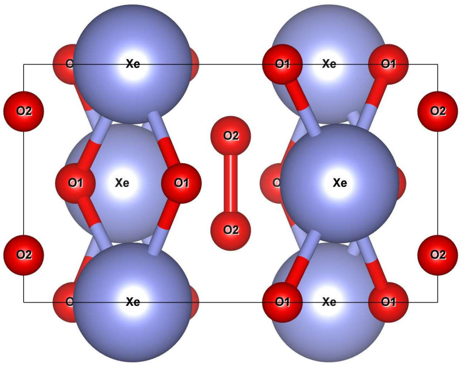

XeO_3, **28**

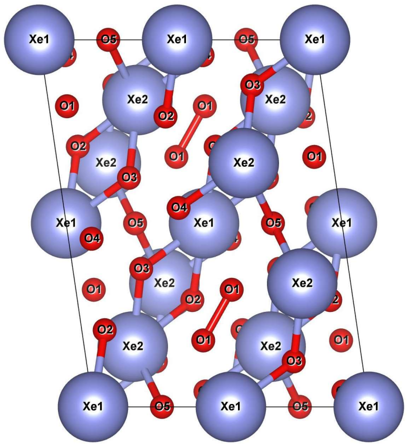

XeO_3, **29**

Figure S12: Structures and numbering of the case studies from Table S13.

Table S13: Results of the EOS analysis for xenon fluorides. Assigned OS, occupation of the frontier eff-AOs ($\lambda_{LO}/\lambda_{FU}$) for each center (overall frontier eff-AOs marked in bold), and reliability index R(%) of the assignation.

ID Molecule	Atom/Fragment	EOS	$\lambda_{LO}/\lambda_{FU}$	Partial Charge	R(%)	Reference OS
**(30) XeF_2**	Xe	**+2**	0.948/**0.383**	+1.12	88.9	
	F	**-1**	**0.773**/0.021	-0.56		
**(31) XeF_3**	Xe1	**+2**	0.943/0.335	+1.24	95.3	
	Xe2	**+4**	0.915/**0.344**	+2.33		
	F1	**-1**	**0.796**/0.023	-0.61		
	F2	**-1**	0.808/0.027	-0.58		
	F3	**-1**	0.812/0.028	-0.59		
**(32) XeF_4**	Xe1	**+4**	0.911/**0.334**	+2.36	97.8	
	F1	**-1**	**0.811**/0.028	-0.59		
	F2	**-1**	0.814/0.027	-0.59		

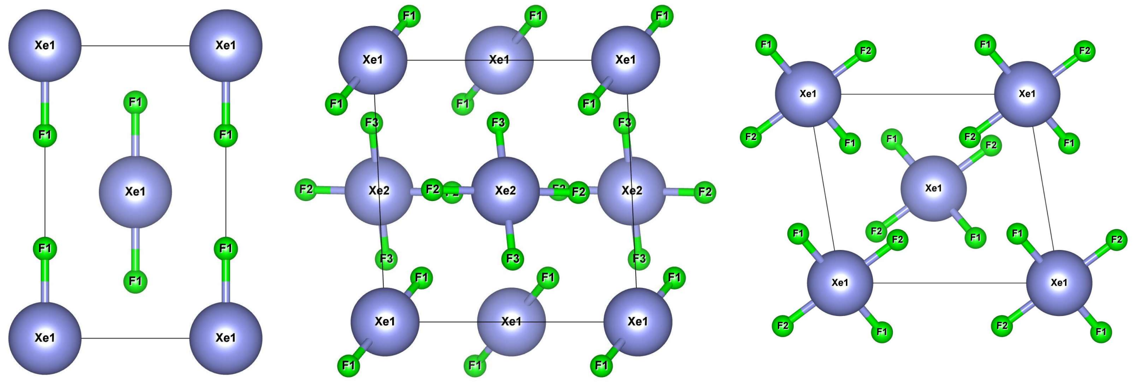

XeF_2, **30** XeF_3, **31** XeF_4, **32**

Figure S13: Structures and numbering of the case studies from Table S14.

Table S14: Results of the EOS analysis for MgXe. Assigned OS, occupation of the frontier eff-AOs ($\lambda_{LO}/\lambda_{FU}$) for each center (overall frontier eff-AOs marked in bold), and reliability index R(%) of the assignation.

ID Molecule	Pressure (GPa)	Space Group	Atom/Fragment	EOS	$\lambda_{LO}/\lambda_{FU}$	Partial Charge	R(%)	Reference OS
**(33) MgXe**	200	*Pm-3m*	Xe	**-2**	**0.211**/0.112	-1.40	59.6	
			Mg	**+2**	core/**0.115**	+1.40		

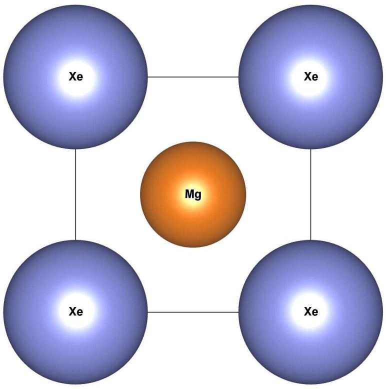

MgXe, **33**

Figure S14: Structures and numbering of the case studies from Table S15.

Table S15: Results of the EOS analysis for HgF_4 compounds obtained at different pressures. Assigned OS, occupation of the frontier eff-AOs ($\lambda_{LO}/\lambda_{FU}$) for each center (overall frontier eff-AOs marked in bold), and reliability index *R*(%) of the assignation.

ID Molecule	Pressure (GPa)	Hg-F distance (Å)	Atom/Fragment	EOS	$\lambda_{LO}/\lambda_{FU}$	Partial Charge	*R*(%)	Reference OS
**(34) HgF_4**	50	1.958	Hg	+4	0.952/**0.684**	1.76	56.3	+4
			F	**-1**	**0.747**/0.028	-0.44		-1
**(35) HgF_4**	100	1.935	Hg	+4	0.947/**0.675**	1.80	57.8	+4
			F	**-1**	**0.753**/0.031	-0.45		-1
**(36) HgF_4**	200	1.893	Hg	+4	0.940/**0.663**	1.86	59.7	+4
			F	**-1**	**0.760**/0.034	-0.47		-1

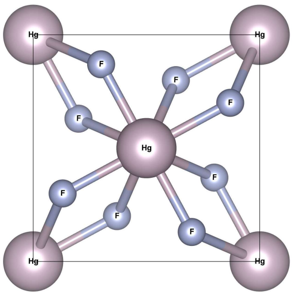

HgF_4, **34**

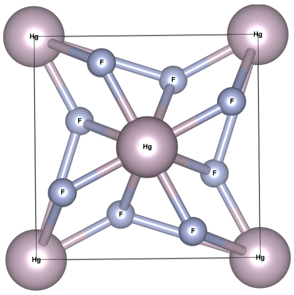

HgF_4, **35**

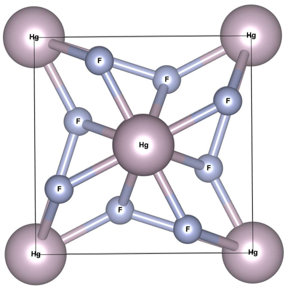

HgF_4, **36**

Figure S15: Structures and numbering of the case studies from Table S16.

Table S16: Results of the EOS analysis for compounds exhibiting homonuclear bonds. Assigned OS, occupation of the frontier eff-AOs ($\lambda_{LO}/\lambda_{FU}$) for each center (overall frontier eff-AOs marked in bold), and reliability index R(%) of the assignation.

ID Molecule	Pressure (GPa)	Space Group	Atom/Fragment	EOS	$\lambda_{LO}/\lambda_{FU}$	Partial Charge	R(%)	Reference OS
**(37) CsH_3**	50	*Cmmm*	Cs	+1	core/**0.041**		100	
			H_3	**-1**	**0.813**/0.022			
	50	*Cmmm*	Cs	+1	core/0.041		67.9	
			H1	**-1**	**0.604**/0.016			
			H2	+1	core/**0.426**			
**(38) CsF_3**	0	*R3m*	Cs	+1	core/0.015		91.3	
			F_3	**-1**	**0.701**/**0.288**			
	0	*R3m*	Cs	+1	core/0.015		66.1	
			F1	+1	0.971/**0.540**			
			F2	-1	**0.701**/0.016			
**(39) CsF_5**	10	*C2/c*	Cs	+1	core/0.072		100	
			F_5	**-1**	**0.779**/**0.154**			
	10	*C2/c*	Cs	+1	core/0.072		56.3	
			F1	**-1**	0.733/0.026			
			F2	+1	0.916/**0.524**			
			F3	**-1**	**0.589**/0.029			
	10	*C2/c*	Cs	+1	core/0.072		100	
			F1	**-1**	**0.733**/0.026			
			F2-F3	**0**	0.927/**0.189**			
**(40) LiN_5**	0	*$P2_1$*	Li	+1	core/**0.019**		100	
			N_5	**-1**	**0.960**/0.017			
	0	*$P2_1$*	Li	+1	core/0.019		50	
			N1	+1	0.559/**0.540**			
			N2	**-1**	0.551/0.445			
			N3	**-1**	**0.540**/0439			
			N4	+1	0.547/0.531			
			N5	**-1**	0.547/0.441			

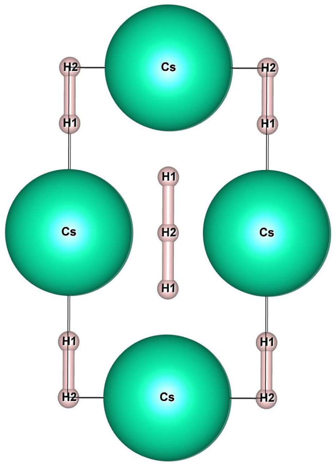

CsH_3, **37**

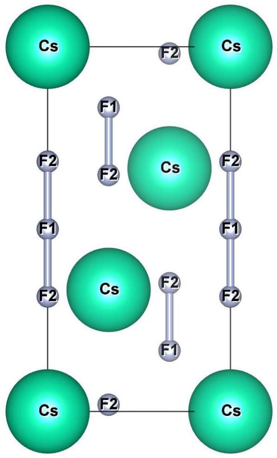

CsF_3, **38**

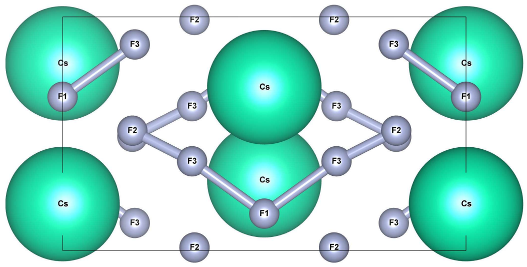

CsF_5, **39**

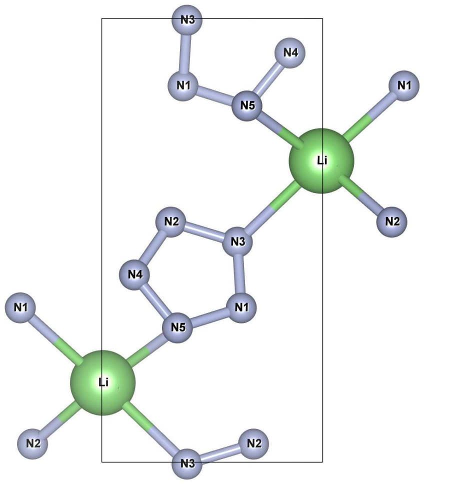

LiN_5, **40**

Figure S16: Structures and numbering of the case studies from Table S17.

References

1. M. Marqués, G. J. Ackland, L. F. Lundegaard, G. Stinton, R. J. Nelmes, M. I. McMahon, *Phys. Rev. Lett.* **2009**, *103*, 115501.
2. T. Bi, E. Zurek, *Chem. Eur. J.* **2021**, *27*, 14858-14870.
3. Q. Zhu, D. Jung, A. Oganov, *et al*. *Nature Chem*. **2013**, *5*, 61-65.
4. M. Miao, X. Wang, J. Brgoch, F. Spera, M. G. Jackson, G. Kresse, H. Lin, *J. Am. Chem. Soc.* **2015**, *137*, 14122-14128.
5. J . Botana, X. Wang, C. Hou, D. Yan, H. Lin, Y. Ma, M. Niao, *Angew. Chem. Int. Ed.* **2015**, *54*, 9280-9283.
6. A. Shamp, J. Hooper, E. Zurek, *Inorg. Chem*. **2012**, *17*, 9333-9342.
7. Q. Zhu, A. Oganov, Q. Zeng, *Sci. Rep.* **2015**, *5*, 7875.
8. F. Peng, Y. Yao, Y. Liu, Y. Ma, *J. Phys. Chem. Lett.* **2015**, *12*, 2363-2366.
